# Gliosarcoma vs. glioblastoma: a retrospective case series using molecular profiling

**DOI:** 10.1186/s12883-021-02233-5

**Published:** 2021-06-23

**Authors:** Christopher Dardis, David Donner, Nader Sanai, Joanne Xiu, Sandeep Mittal, Sharon K. Michelhaugh, Manjari Pandey, Santosh Kesari, Amy B. Heimberger, Zoran Gatalica, Michael W. Korn, Ashley L. Sumrall, Surasak Phuphanich

**Affiliations:** 1 Department of Neurology, Barrow Neurological Institute, Phoenix, AZ, USA; 2 School of Medicine, Creighton University, Phoenix, AZ, USA; 3 Barrow Brain Tumor Research Center, Department of Neurosurgery, Barrow Neurological Institute, Phoenix, AZ, USA; 4 Precision Oncology Alliance, Caris Life Sciences, Phoenix, AZ, USA; 5 Fralin Biomedical Research Institute, Virginia Tech Carilion School of Medicine, Roanoke, VA, USA; 6 Department of Medical Oncology, West Cancer Center, University of Tennessee Health Science Center, Germantown, TN, USA; 7 Pacific Neuroscience Institute and Department of Translational Neurosciences and Neurotherapeutics, John Wayne Cancer Institute, Santa Monica, CA, USA; 8 Simpson Querry Biomedical Research Center, Department of Neurosurgery, Feinberg School of Medicine, Northwestern University, Chicago, IL, USA; 9 Department of Pathology, University of Oklahoma Health Sciences Center, Oklahoma City, OK, USA; 10 Department of Medical Oncology, Levine Cancer Institute, Atrium Health, Charlotte, NC, USA; 11 Department of Medicine, Samuel Oschin Comprehensive Cancer Institute, Cedars-Sinai Medical Center, Los Angeles, CA, USA

**Keywords:** Gliosarcoma, Glioblastoma, Molecular profiling, Pan-cancer analysis, Epithelial-to-mesenchymal transition, Immuno-evasion

## Abstract

**Background:**

Gliosarcoma (GS) refers to the presence of mesenchymal differentiation (as seen using light microscopy) in the setting of glioblastoma (GB, an astrocytoma, WHO Grade 4). Although the same approach to treatment is typically adopted for GS and GB, there remains some debate as to whether GS should be considered a discrete pathological entity. Differences between these tumors have not been clearly established at the molecular level.

**Methods:**

Patients with GS (*n*=48) or GB (*n*=1229) underwent molecular profiling (MP) with a pan-cancer panel of tests as part of their clinical care. The methods employed included next-generation sequencing (NGS) of DNA and RNA, copy number variation (CNV) of DNA and immunohistochemistry (IHC). The MP comprised 1153 tests in total, although results for each test were not available for every tumor profiled. We analyzed this data retrospectively in order to determine if our results were in keeping with what is known about the pathogenesis of GS by contrast with GB. We also sought novel associations between the MP and GS vs. GB which might improve our understanding of pathogenesis of GS.

**Results:**

Potentially meaningful associations (*p*<0.1, Fisher’s exact test (FET)) were found for 14 of these tests in GS vs. GB. A novel finding was higher levels of proteins mediating immuno-evasion (PD-1, PD-L1) in GS. *All* of the differences we observed have been associated with epithelial-to-mesenchymal transition (EMT) in other tumor types. Many of the changes we saw in GS are novel in the setting of glial tumors, including copy number amplification in *LYL1* and mutations in *PTPN11*.

**Conclusions:**

GS shows certain characteristics of EMT, by contrast with GB. Treatments targeting immuno-evasion may be of greater therapeutic value in GS relative to GB.

**Supplementary Information:**

The online version contains supplementary material available at (10.1186/s12883-021-02233-5).

## Background

GB and GS are defined by the WHO as Grade 4 astrocytomas; GS accounts for a small subset of GB. GS is distinguished from GB on the basis of features readily distinguished via light microscopy i.e. the presence of a sarcomatous component in the tumor.

The WHO maintains defines GS as follows [1]:

a variant of IDH-wildtype glioblastoma characterized by a biphasic tissue pattern with alternating areas displaying glial and mesenchymal differentiation

The WHO also notes that

expression of SNAI2, TWIST, MMP2 and MMP9 is characteristic of mesenchymal areas, suggesting …epithelial to mesenchymal transition (EMT) may play a role.

The molecular changes seen in GS were summarized as part of a review of the literature to date on GS in 2010, comprising 219 cases [2]. The authors noted the lower frequency of *EGFR* copy number amplification (CNA) in GS (c. 8%) vs. GB (up to 50%). They also note that prior case series assessing both the glial and the sarcomatous elements of GS found both elements shared common genetic and chromosomal alterations of the type typical of GB. Such findings indicate a common clonal origin for both the glial and the sarcomatous elements seen with light microscopy.

Molecular profiling (MP) means determining whether a (potentially) pathogenic molecular change is present in a tumor. Such changes can be assessed at the level of DNA, RNA, protein or chromosome. In high-throughput settings, for the sake of efficiency, such MP is typically performed as a standard panel of tests [3]. In clinical oncology, this often takes the form of a pan-cancer panel i.e. a panel with the same set of molecular tests (MTs), no matter the type of tumor being analyzed. The variety of methods which may be used to demonstrate such changes is illustrated by those used in our case series (Table 1). This list is not exhaustive; additional methods may be employed depending on the context. In clinical practice, the commercial availability of MP has lead to the development of personalized treatments. For example, MP has been approved by the FDA to look for genetic changes which have specific, targeted treatments, particularly in melanoma and NSCLC, colorectal, breast and ovarian cancer [4]. In the academic setting, a pre-selected set of MTs may be used to characterize a particular tumor and it’s pathogenesis.

**Table 1:** Methods used for molecular profiling, sorted by number of tests

Abbreviation	Method	Molecule	*n*	Examples	
				NSD	From Table 3
NGS	next generation sequencing	DNA	599	*PTEN*, *TP53*	*IDH1*, *IDH2*, *PTCH1*
CNA	copy number amplification	DNA	444	*ALK*, *BRAF*	*CALR*, *LYL1*, *EGFR*
F-RNA	fusion gene present	RNA	54	*EGFRvIII*, *BRAF*	*NTRK1*
IHC	immunohistochemistry	protein	28	ALK, EGFR	PD-1, PD-L1
SS	Sanger sequencing	DNA	9	*BRAF1*, *KIT*	
FISH	fluorescence in-situ hybridization	DNA	6	del(1p/19q), *HER2/neu*	
CISH	chromogenic in situ hybridization	DNA	5	*EGFR*, *HER2/neu*	
RFLP	restriction fragment length polymorphism	DNA	3	*EGFR T790M*	
FA	fragment analysis	DNA	2		*EGFRvIII*
FV	fusion variant	RNA	2	*EGFR*, *MET*	
FFA	fusion or fragment analysis		1		*EGFRvIII*
HS	H score (immunohistochemistry score)	protein	1	EGFR	
P	pyrosequencing	DNA	1	MGMTpm status	
MSI	microsatellite instability	DNA	1		

Some of these abbreviations are used in Tables 3 and 5. Examples of DNA/ RNA/ proteins assessed are given for each method

Abbreviations: *n* Number of tests using this method, *NSD* Test was not significantly different (GS vs. GB), *MGMTpm* MGMT promoter methylation

As discussed in detail below, the studies to date which have used MP to assess GS vs. GB have been of this academic type. They examined relatively small numbers of specimens and employed a variety of techniques, including various approaches to sequencing (whole-exome sequencing, Sanger sequencing, single nucleotide polymorphisms (SNiPs), next-generation sequencing (NGS) and RNA sequencing with gene-expression profiling) as well as copy number variation (CNV). As with our case series, these previous studies appear to have had no definite hypothesis in mind when the panel of MTs was constructed. Unlike our series, other prior studies have used MP designed specifically to examine GS vs. GB. We reasoned that using a pan-cancer panel of MTs would add to our understanding of the pathogenesis of GS as many of the MTs in our panel have not previously been examined in this context. Thus, we expected to confirm some of the existing results in this field as well as adding some novel findings. As the largest case series to date to describe GS using MP, we envisaged that our sample size would make our results more robust than some of the work to date in this area.

## Methods

### Aim, setting and design

Our primary main aim was to see whether any of the tests in our pan-cancer panel more frequently altered in GS vs. GB or vice versa. A secondary aim was to describe GS vs. GB in terms of the additional data available to us i.e. differences in demographics, the anatomic site of the tumor and the features present via light microscopy.

The data we used for this analysis was provided by Caris Life Sciences, who performed the MP. Our analysis was performed retrospectively; the data was not collected specifically to address differences in GS vs. GB. There were 264 cases of GB where the diagnosis was made on the basis of biopsy alone; due to the possibility that some of these may have had a sarcomatous element which was not sampled on biopsy, we omitted these cases from further analysis. This left 48 cases of GS and 1229 cases of GB. The diagnosis of GS or GB was made by the Pathologist at the treating institution. GS constituted 4% (48/1229) of the cases, which is broadly in keeping with the more typical rate of 2% usually given [5]. The relative rarity of GS vs. GB can make quantitative comparisons more challenging. In order to maximize our sample size, and as GS is so rare relative to GB, we chose to include 139/1229 cases (11%) where the tumor was recurrent, a proportion which was similar for GB and GS. For the same reason, we included cases with mutations of *IDH1* or *IDH2* as it was not evident to us that GS should a priori be considered as IDH-wildtype, as per the WHO definition above.

The data covered the period 2009–2014. Specimens came from 79 institutions across 25 states in the USA. These included tertiary academic medical centers as well as smaller regional cancer centers. MP was not performed for every case of GS or GB seen at a participating institution during this period of time. A decision to perform MP was typically made both to look for results which could affect clinical decision making (particularly to look for molecularly-targeted treatments) and to improve our understanding of these tumor types. The MP comprised 1153 MTs which made up a standardized pan-cancer panel (i.e. the MP did not depend on the type of cancer being profiled). This used a variety of methods to analyze each specimen, as shown in Table 1. Further details of the methods, including technical specifications and accuracy, can be found on the Caris website [6]. All MTs were reported as a dichotomous outcome i.e. “positive” (indicating a pathological change such as a mutation or amplification of DNA) or “negative”. Using a pan-cancer panel such as this had the advantage of including many tests not normally assessed in GB, e.g. mutations in *SHP-2*, leading to the possibility of novel observations. The corollary is that many of the MTs were not likely to be relevant to either GB or GS, e.g. testing for *ALK* mutations as typically seen in NSCLC. Indeed, most tests showed no positive cases in either GS or GB (904/1153, 78%), or no positive results in any cases of GS (192/1153, 17%). Details of these MTs, with hyperlinks to the relevant entry in the NCBI’s Gene database, can be found in the Supplemental Data. Only 54/1153 (5%) of the MTs had positive results in at least one case of both GS and of GB. Not every test could be performed on every specimen, although results for the majority were available (median of 947 tests per specimen). The only MT that was reported as a continuous variable was “tumor mutational load” (TML, or “tumor mutational burden”). TML was classified by Caris as “high” (vs. “low”) when there were ≥17 mutations per mega (1×10^6^) base-pairs (MB) of DNA, irrespective of the type of tumor being analyzed. As GB is known to have a lower TML than e.g. melanoma, we checked whether an alternative cutpoint might be more useful in distinguishing GS from GB.

In addition to the results of the MP, the age, gender and the anatomical site of the tumor were recorded for each case. Characteristics of the tumor visible with light microscopy were recorded in many cases as shown in Table 2. However, the reporting of such features was not required, nor was it standardized. The most problematic in this regard was “tumor location”, which initially comprised 36 categories. We derived a simpler summary of location by grouping these into 7 categories. For tumors affecting the brain, this was coded as “brain” in some cases; others had enough detail to specify one or more lobes. A separate variable was used to record three cases where the tumor was metastatic to sites beyond the nervous system (intradural, to a lymph node or to subcutaneous tissue, respectively; all of these were GB). As this data was not being collected in order to predict prognosis, other information which would have been useful in this regard was not available such as the type of surgery, performance status of the patient, their treatment and the time to progression. Nonetheless, the absence of such information did not affect our aim of assessing the molecular differences between these tumor types.

**Table 2:** Pathological features; sorted by odds ratio (OR)

Feature	*p* (FET)	nGS	pGS	nGB	pGB	OR
Necrosis reported?	0.62	2/48	4.2%	31/1181	2.6%	1.6
Tumor was recurrent or residual?	0.49	7/48	15%	132/1181	2.6%	1.6
Oligodendroglial features?	0.63	0/48	0%	18/1181	1.5%	0
Intratumoral hemorrhage?	1	0/48	0%	7/1181	0.6%	0
Features of giant cell GB?	1	0/48	0%	7/1181	0.6%	0
Features of epithelioid GB?	1	0/48	0%	6/1181	0.5%	0
Gemistocytic?	1	0/48	0%	4/1181	0.3%	0
Features of small-cell GB?	1	0/48	0%	6/1181	0.5%	0
Treatment effect/ radiation necrosis?	0.68	0/48	0%	14/1181	1.2%	0
Fibrillary?	1	0/48	0	1/1181	0.09%	0

Abbreviations: *p* (FET) Probability (*p* value, Fisher’s exact test), *nGS* Number of cases with the feature in gliosarcoma (+ve/total), *pGS* Percentage in cases of gliosarcoma, *nGB* Number of cases with the feature in glioblastoma (+ve/total), *pGB* Percentage in cases of glioblastoma, *OR* Odds ratio = pGS/pGB

### Statistical analysis

The data was scrubbed using Gnumeric (RRID:SCR 018462). The complete data-set used is provided with the Supplemental Data accompanying this article (gb-gs.xlsx). Data analysis was performed using R (RRID:SCR 001905) and is also provided (gs-gb.pdf) [7]. This analysis includes some additional tests and results that, for reasons of space, are not included in the main article.

All variables were considered as either nominal/categorical or continuous. Continuous variables were summarized as median (range). The only variables which might have been considered as ordinal/ordered were “extent of resection” and “tumor location”. We saw no reason to designate these as ordinal, as these were assessed only in tests of correlation (rather than modeling e.g. via regression).

Our analysis proceeded through the steps of descriptive statistics, tests for significant correlation/association, testing for significant classifiers (as GS vs. GB) and testing for significant predictors via regression modeling, with the outcome being GS (vs. GB). As exploratory or hypothesis-generating work, we made no correction for multiple hypothesis-testing/multiple comparisons [8].

We looked at standard descriptive statistics appropriate to the variable type. Continuous variables were assumed to be normally distributed, given the sample size. Thus correlation between continuous variables was assessed with *t*-tests (one or two-sided, as appropriate). For nominal variables, we first inspected was contingency tables. (Where such tables show perfect classification (at least one cell has no observations), statistics are of course not meaningful). For 2×2 tables, we used the odds ratio (OR) as a measure of effect size, given with a 95% CI in Table 3. To test significance, we used Fisher’s exact test (FET); this was two-sided as this was exploratory work. We chose FET in preference to the chi-squared test as many of these tables had at least one cell with a count of less than 5. For tables larger than 2×2 we used the chi-squared statistic to check whether the observations in the tables were unevenly distributed.

**Table 3:** Molecular tests with a significant (*p*<0.1) association with GS; sorted by OR

Test	Method	NCBI	*p* (FET)	nGS	pGS	nGB	pGB	OR	95% CI OR	EMT
**More positive results in GS vs. GB**										
*CALR*	CNA	CALR	0.038	1/24	4.2%	0/603	0%	*∞*	0.64 — *∞*	[9]
*NTRK1*	F-RNA	NTRK1	0.074	1/22	4.5%	1/563	0.2%	25	0.33 — 2100	[10]
*LYL1*	CNA	LYL1	0.075	1/24	4.2%	1/603	0.2%	25	0.32 — 2000	[11]
*PTCH1*	NGS	PTCH1	0.081	1/25	4%	1/582	0.2%	24	0.30 — 1900	[12]
*IDH2*	NGS	IDH2	0.080	1/27	3.7%	1/631	0.2%	23	0.30 — 1900	[13]
*SHP-2*	NGS	PTPN11	0.080	3/47	6.4%	21/1046	2%	3.2	0.61 — 12	[14, 15]
*NF1*	NGS	NF1	0.019	8/25	32%	81/587	2.3%	2.7	1.1 — 7.5	[16]
PD-L1	IHC**	CD274	0.0057	15/42	36%	152/902	17%	2.1	1.3 — 5.5	[17, 18]
PD-1	IHC	PDCD1	0.014	20/26	77%	211/408	52%	1.5	1.2 — 9.6	[19]
**Fewer positive results in GS vs. GB**										
*EGFRvIII*	FFA		0.02	2/38	5.3%	143/706	20%	0.27	0.03 — 0.88	
*EGFRvIII*	FA		0.06	1/24	4.2%	71/349	20%	0.21	0.004 — 1.1	
*EGFR*	CNA	EGFR	0.0008	2/27	7.4%	231/623	37%	0.20	0.02 — 0.55	[20]
*EGFR*	NGS		0.011	0/47	0%	112/1044	11%	0	0 — 0.69	
*IDH1*	NGS	IDH1	0.046	0/47	0%	87/1047	8.3%	0	0 — 0.92	

Abbreviations: *Test* Common name, *Method* See abbreviations in Table 1; ** = using SP142 antibody, *NCBI* Hyperlink to NCBI GeneID, given by Official Name, *p (FET)* probability (*p* value, Fisher’s exact test), *nGS* Number of positive results in cases of gliosarcoma (+ve/total), *pGS* Percentage of positive results in cases of gliosarcoma, *nGB* Number of positive results in cases of glioblastoma (+ve/total), *pGB* Percentage of positive results in cases of glioblastoma, *OR* Odds ratio = pGS/pGB, *95% CI OR* 95% confidence interval for OR, *EMT* Reference(s) supporting the role of gene/protein in epithelial-to-mesenchymal transition (EMT)

**Fig. 1:**
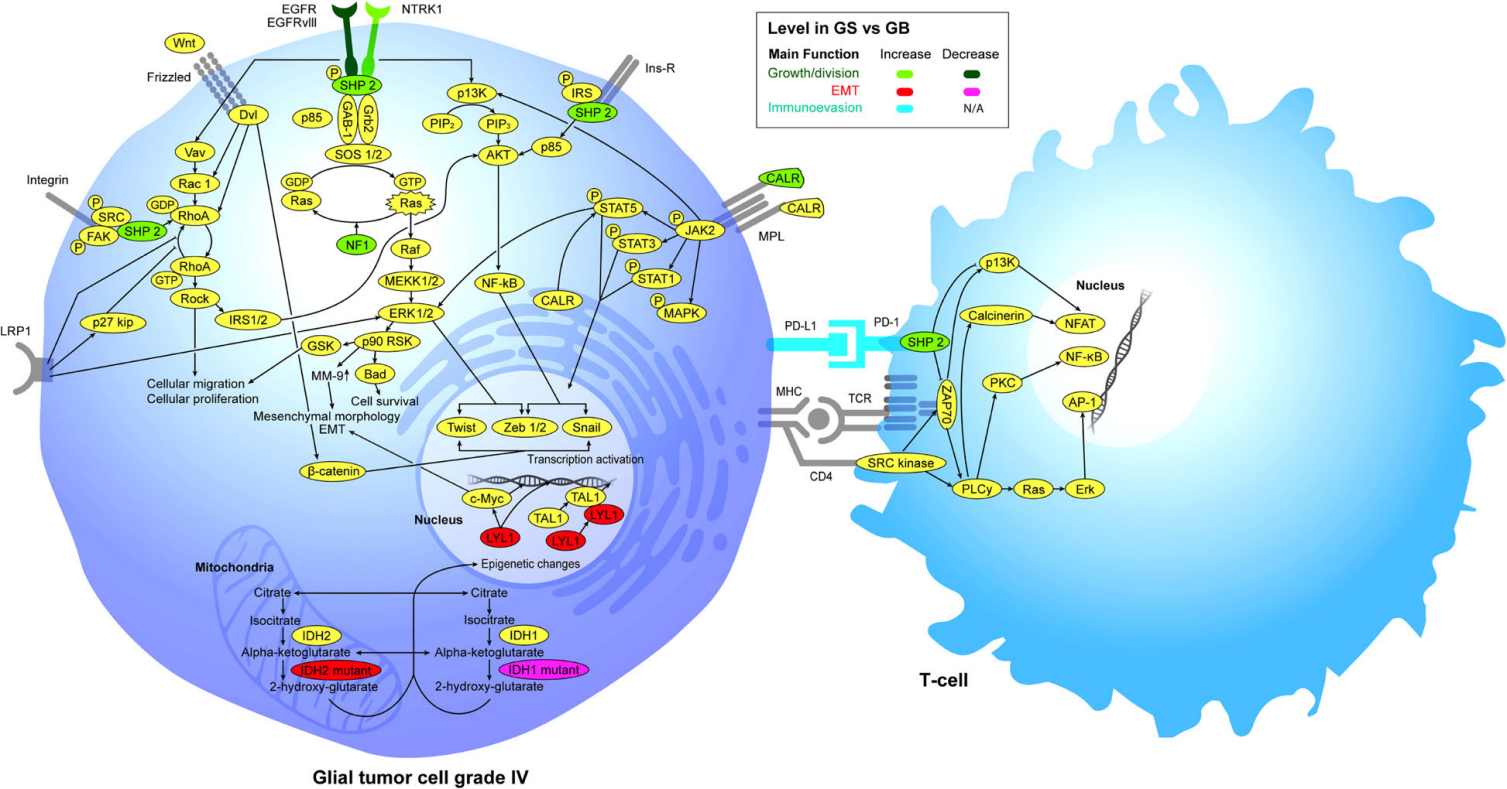
Proteins and relevant pathways altered in GS vs. GB. The Official Name for SHP-2 is PTPN11. This shows all of the proteins in Table 3, except PTCH1. This latter is omitted for reasons of space and clarity as it’s downstream pathway is largely unconnected to the other proteins shown here

Significance testing and robustness can be problematic in the context of a study such as ours with a relatively small sample size and relatively few significant tests of correlation [21]. We initially considered reporting only those results with *p*<0.05. However, on tests of correlation, this lead to just 8 “significant” correlations (of a MT with GS vs. GB). We thus broadened our scope to look at associations with *p*<0.1, which lead to an additional 6 correlations. When reflecting mechanistically on how these might all be related (Fig. 1), we felt that all 14 correlations were worth reporting.

As a simple check of the robustness of our results, we assessed whether the 95% CI of the OR contained ’1’, as shown in Table 4. If so, the estimate of the effect size should be interpreted cautiously. For the *p* values from FET, we checked whether our result would remain significant if one observation were added to or removed from (where possible) each cell in the contingency table used to calculate this *p* value. For example, *CALR* showed CNA in the 1/24 cases of GS for which the MT was reported (vs. 0/603 cases in GB, shown in Table 3) and thus was significant at *p*<0.05. However, a single case is not necessarily indicative of a trend. The 95% CI of the OR contains ’1’ and the significance would of course have been lost if without this single case, even with a more generous *p*<0.1.

**Table 4:** Robustness of *p* values; sorted by *p* (Fisher’s exact test)

Test	Method	*p* (FET)	*p*<0.05	robust		
				OR	*p*<0.1	*p*<0.05
*EGFR*	CNA	0.0008	*	*	*	*
PD-L1	IHC	0.0057	*	*	*	*
*EGFR*	NGS	0.011	*	*	*	
PD-1	IHC	0.014	*	*	*	*
*NF1*	NGS	0.019	*	*	*	
*EGFRvIII*	FFA	0.020	*	*	*	
*CALR*	CNA	0.038	*			
*IDH1*	NGS	0.046	*	*		
*EGFRvIII*	FA	0.060				
*NTRK1*	F-RNA	0.074				
*LYL1*	CNA	0.075				
*SHP-2*	NGS	0.0877				
*IDH2*	NGS	0.081				
*PTCH1*	NGS	0.081				

* indicates significance. Robustness was assessed as follows: OR = 95% CI for odds ratio (in Table 3) excludes ’1’; *p* values were examined to ensure they remained stable after adding/removing one observation from each cell in the contingency table from which the original *p* value was calculated

To find the variables which allowed for the most accurate classification of each case (as GS or GB) we used recursive partitioning [22]. For the 14 variables found to be potentially meaningful via FET, we looked at models fit with logistic regression (all containing an intercept term). Effect size for the variable was again expressed as the OR and significance was tested using the Wald test. We assessed their importance in a model including all 14 of these variables, as well as in 14 uni-variable models. We then sought those variables which retained their significance in models with small numbers of predictors.

## Results

### Molecular tests

As above, there were 54 MTs with potentially meaningful results for FET. Table 3, shows the 14 of these with *p*<0.1. These reflect a variety of alterations, primarily assessed via DNA, with mutations (via NGS) in 6 cases and CNA in 3 cases. The only cases where proteins were assayed directly, rather than DNA or RNA, were PD-L1 and PD-1. The function of these genes/proteins is discussed in the Discussion below. The results in Table 3 are sorted by OR (proportion of positive cases/total in GS vs. GB). Table 3 also gives the ratios from which the OR is calculated i.e. for both GS and GB we give the number of positive results/ number of specimens where the MT was done. We provide hyperlinks in this Table to the NCBI for to further information on the relevant gene as well as references to literature indicating the role of the gene/protein in EMT in other tumor types. As *EGFR*/*EGFRvIII* appears 4 times in Table 3, we provide a single hyperlink and reference for these MTs.

As above, not every test could be performed on every specimen, so the total number of cases varies for each test (e.g. in the first row, *CALR*, the denominator indicating the number of instances of where this test was done for case of GS is 24 vs. 22 in the next row, *NTRK1*). The first 9 cases have an OR >1 i.e. there are more positive results in cases of GS vs. GB. In the case of *CALR* the OR is given the value of infinity as there were no cases of this CNA occurring in GB. The first 5 MTs in this table were positive in just 1 case of GS and 0—1 cases of GB and so should be interpreted cautiously. Hence, these tests all have very wide 95% CIs for the OR, which includes the value of ’1’ in each case, indicating that the value for the OR is not particularly robust and would change substantially with the addition of a small number of positive cases, whether the tumor was GS or GB.

The tests with the greatest number of positive cases in GS were *NF1*, PD-L1 and PD-1. As all of these showed pathogenic changes in >30% of cases of GS, we can be more certain that these were relatively common in GS. For the other 11 MTs, pathological results were seen in ≤3 cases of GS. In these cases, we acknowledge that with such small numbers we cannot definitively infer their importance in characterizing GS. However, we include these MTs in part due to the relatively large number of negative results seen in cases of GB relative to GS.

The MTs with *fewer* positive results in GS (OR <1) all had a reasonably high percentage of positive cases in GB (>8*%* in relatively large numbers of cases). Thus, the low numbers of positive cases in GS are of less importance here. All of these MTs relate to *EGFR*, with the exception of *IDH1*. *EGFRvIII* was less common in GS, as was CNA of *EGFR* and mutations in *EGFR* via NGS. There were no cases of *IDH1* mutation via NGS in GS, as may have been expected. However *IDH2* did show a mutation in 1/27 cases of GS, indicating that such mutations do not preclude the diagnosis of GS.

These same 14 MTs are shown in Table 4, sorted by *p* value (via FET). The first 8 of these all show *p*<0.05, with the first two having *p*<0.01. We also indicate whether the 95% CI for the OR for each test excludes the value of ’1’; if so, this supports the view that the effect size did not arise by chance. Table 4 provides an indication of the “robustness” of these *p* values. When assessing the effect of adding or removing a single positive or negative result for either GS or GB, three of the four MTs with the lowest original *p* values retained their significance at *p*<0.05. These were *EGFR* (CNA, more common in GB) as well as PD-L1 and PD-1 (positive via IHC in more cases of GS). By contrast, the final 6 tests in this table, with *p* values in the range 0.05—0.10, were all sensitive to the effect of adding/removing a single result.

Using uni-variable logistic regression, 9 of the 14 MTs above showed *p*<0.05 for the effect of the MT (Table 5). Regarding the effect size (OR) for these MTs, it is reassuring that the 95% CIs do not contain ’1’ and that the ORs were relatively stable vs. Table 3. There was a slight disparity in the order of MTs in Table 4 vs. Table 5. This is accounted for by the significance of the intercept term in logistic modeling, which in all cases was far more significant than the predictor (see Additional Files). This is also evident in the best two-variable model, where the significance of the intercept term is striking but both predictors are relatively marginal (0.05<*p*<0.1). Recursive partitioning (to classify the pathology as GS vs. GB) confirmed the usefulness of the following MTs, in order of importance: *CALR* CNA, *NTRK1* fusion, PD-L1 via IHC and *IDH2* mutation. None of the other variables in Table 3 were useful in recursive partitioning (see Additional Files for further details and plots relevant to this analysis).

**Table 5:** Logistic regression models, sorted by *p* value

Model term	Method	*p* (Wald)	OR	95% CI
**Uni-variable models with*p*<0*.*05**				
PD-L1	IHC	0.0025	2.7	1.7 — 4.8
*EGFR*	CNA	0.0069	0.14	0.03 — 0.54
*NF1*	NGS	0.015	2.9	1.4 — 6.0
PD-1	IHC	0.017	3.1	2.2 — 5.5
*NTRK1*	F-RNA	0.022	27	2.0 — 507
*LYL1*	CNA	0.022	26	1.9 — 498
*IDH2*	NGS	0.026	24	1.8 — 465
*PTCH1*	NGS	0.026	24	1.3 — 159
*EGFRvIII*	FFA	0.04	0.22	0.06 — 0.95
**Best two-variable model**				
Intercept		<0.001	0.04	0.01 — 0.15
*EGFR*	CNA	0.084	0.26	0.06 — 1.1
PD-1	IHC	0.072	4.1	1.0 — 17.3

Uni-variable models with *p*<0.05; values for *p* and OR not shown for the intercept term in these models. The best two-variable model is also shown

Abbreviations: *Method* = See abbreviations in Table 1, *p* Probability (*p* value, Wald statistic), *OR* Odds ratio, *95% CI* 95% confidence interval for OR

The connections between the genes/proteins in Table 3 are illustrated in Fig. 1, which also includes some of the proteins that act as intermediaries in these pathways. In this figure, some proteins are colored to reflect their primary role in cell growth/division, although many also have downstream signaling effects mediating EMT e.g. CALR, SHP-2 (PTPN11). It is particularly striking that SHP-2 is a downstream mediator of the effects of EGFR, NTRK1 and of PD-1, all of which were up-regulated in GS vs. GB. This provides further rationale for including results for SHP-2 in Tables 3 and 4, even though 0.05<*p*<0.1 for the correlation of this MT with GS vs. GB.

There was a tendency for TML to be higher in GS (mean 12.2 vs. 7.8 in GB), although this was not significt (*t*-test, one-sided, *p*=0.15). We found a cutoff TML of ≥8 per MB to be the best classifier of GS vs. GB, as opposed to the standard cutoff of ≥17 per MB which was reported with this panel of MTs. Using this lower cutoff for TML also did not reach significance (FET).

### Other variables

As shown in Table 6, patients with GB were older; age 57 (4-90) vs. 54 (26-78) for GS; *t*-test, one-sided, *p*=0.04. There was a tendency for males to account for a greater proportion of those with GB, 62%, vs. GS, 52%; OR 0.62; FET, one-sided, *p*=0.1. As may be expected, there was no significant difference in the proportion of cases of GS vs. GB in terms of the year the specimen was collected or of the Institution or State where the material was collected (see Additional Files).

**Table 6:** Demographics and site of tumor

	*n*	GS	GB	*p*	Statistical test
Age	1229	55 (26-78)	58 (4-90)	0.04	*t*-test; GB older
Gender: male vs. female	1229	25/48 (52%)	733/1181 (62%)	0.1	FET; higher % in GB
**Site**					
Brain vs. spinal cord	1225	0/47 (0%)	4/1178 (0.3%)	0.85	FET; higher % in GB
Brain: supra- vs. infra-tentorial	1221	0/47 (0%)	11/1437 (0.9%)	0.65	FET; higher % in GB

Values for GB and GS are given as median (range) or as a fraction (%), as appropriate.

Abbreviations: *n* Number of cases, *p* Probability (*p* value), *FET* Fisher’s exact test

The anatomic site of the tumor was recorded in all cases. We note some minor differences here, although none of these were significant (FET). There were no cases of GS reported in the spinal cord (vs. 4/1178 (0.3%) with GB). There were also no cases of GS reported as “infra-tentorial” (affecting the brainstem and/or cerebellum) vs. 11/1174 (0.9%) of cases of GB. No cases of GS were recorded as “bilateral” or “midline” (i.e. arising from centrally-located structures) vs. 12/976 (1.2%) of those with GB. None of the characteristics shown in Table 2 were significantly different between GS and GB (FET). We note that most of these features were not reported in *any* cases of GS.

## Discussion

### Molecular characteristics in our series

Here we discuss the MTs with positive results in our series with respect to the primary function of each protein, as shown in Fig. 1. There is some overlap here as many of the proteins we highlight are classically associated with mitosis but also appear to mediate EMT.

The ability of cancer cells to deactivate the immune system which should be targeting them appears to be a universal property of neoplastic cells, as there appears to be no example to date of a tumor where this property has *not* been recognized. Immuno-evasion has been linked to EMT, particularly in non–small cell lung cancer (NSCLC) [17, 23]. The best understood mechanism by which this occurs involves the ligand protein PD-L1, produced by tumor cells, binding it’s receptor PD-1 on a T-cell (leading to activation of SHP-2, as shown in Fig. 1). This inhibits the cell-killing ability of the T-cell. Increased expression of PD-1 and PD-L1, is an established feature of EMT in a variety of tumor types [17–19]. The role of PD-1/PD-L1 in the pathogenesis of GB and the potential for targeting this pathway has been reviewed [24]. Higher levels of these proteins and higher levels of tumor infiltrating lymphocytes have previously been noted in GS vs. GB in a series including 233 WHO Grade 4 gliomas with 9 cases of GS [25]. Treatments targeting this process have proven clinical value in tumors such as melanoma, NSCLC, head and neck carcinoma as well as some leukemias and lymphomas [26]. Whether targeting PD-L1 in the setting of GB will be similarly effective remains an area of active investigation [27].

As above, the WHO have indicated that EMT may play a role in the pathogenesis of GS, although it remains an open question as to whether EMT is pathognomic of GS. Here, we briefly review this phenomenon as well as it’s relevance to various sub-types of GB before discussing our results in this context. In EMT, adverse conditions in the tumor microenvironment (hypoxia, acidity and starvation) lead to tumor de-differentiation, into what appears to be a mesenchymal phenotype. This involves a change in metabolism from oxidative phosphorylation to glycolysis. The pattern of gene expression is altered, occurring in part through a change in chromatin structure (via methylation and acetylation) as well as through the change in expression of certain non-coding microRNAs (miRs). Many of the genes involved in EMT change their pattern of expression, becoming ‘bivalent’ i.e. allowing their expression to rapidly change in response to stimuli from the local environment. The cancer cells thereby develop a “stem-like” phenotype. EMT is recognized in a variety of tumors of ectodermal origin, including neuro-epithelial tumors. Additional details are available in the form of review articles, both from the broad perspective of normal physiology and embryology as well as in the context of cancer, which appears to develop analogous alterations [28, 29]. Transcription factors (TFs) are key mediators of EMT, particularly SNA1, Twist, NF-kB, ZEB1 and Sox1 [29]. The process is also driven, in part, by the local activity of growth factors, particularly hepatic, epidermal, fibroblast and platelet-derived (PDGF). Reviews of EMT specific to the context of malignant glioma concur with the importance of these key TFs as well as additional miRs [30, 31]. GS has received less attention than other neuroepithelial tumors in regards to EMT, perhaps in part due to it’s relative rarity.

While mesenchymal differentiation remains the most widely accepted explanation for these phenomena, it has been proposed that transition to a *myeloid* phenotype by the tumor provides a better and more encompassing framework for understanding such changes [32]. This epithelial-to-myeloid transition (EMyeT) appears to provide a better explanation for the marked inflammatory reaction to certain cancers as well as their effects on bone (metastases, remodeling). Whether EMyeT provides a better explanation for the pathology of GS than EMT remains to be investigated.

MP can be used to divide GB into sub-types. The number of sub-types will depend somewhat on the investigators preference for “lumping” or “splitting” as well as the MTs used. Consensus has developed that division into four sub-types is paradigmatic, although one tumor may display more than one of these biochemical phenotypes. These are (with their characteristic pathological changes): Classical (EGFR), Mesenchymal (NF1), Proneural (PDGFRA, IDH1) and Neural (neural cell markers) [33]. There remains some debate as to whether some of these have a greater tendency to display a profile characteristic of EMT. The study by Verhaak et al., which proposed the above classification, noted that the Mesenchymal sub-type showed changes reminiscent of the EMT seen in other tumor types, to whit higher activity of mesenchymal and astrocytic markers (CD44 and MERTK). It has since been noted that the Proneural sub-type has a particularly tendency towards EMT and may evolve into the Mesenchymal sub-type; the term Proneural-Mesenchymal transition (PMT) has been proposed to describe this phenomenon [34, 35]. It remains unclear whether GS has a particular tendency to arise in the context of one of these sub-types. As most cases appeared to arise de novo (41/48, 85%, in our series), it may be challenging to determine whether GS has a greater propensity to arise from one of these sub-types of GB.

LYL1 is a transcription factor, which up-regulates expression of angiopoetin-2, the latter being involved in angiogenesis [36]. Increased expression of its co-factor, LMO2, has been noted in GB cells with a stem-like phenotype [11]. As noted above, the stem-like phenotype is recognized to overlap widely with EMT, in GB as with other tumor types [37]. PTCH1 (patched 1) is a cell-surface receptor for the sonic hedgehog (Shh) ligand. Mutations of *PTCH1* have been associated up-regulation of the Shh response and with EMT [12]. Mutations in *IDH1* or *IDH2* lead to the production of D-2-hydroxyglutarate, as shown in Fig. 1. This ‘oncometabolite’ has been associated with EMT in other tumor types, such as colorectal carcinoma [38]. In this setting, EMT is reported to be mediated primarily by up-regulation of the TF ZEB1 [13]. The WHO define GS as an IDH-wildtype astrocytoma and in keeping with this we saw no mutations in *IDH1* in GS in our series (vs. 87/1047, 8.3%, in GB). However, *IDH2* mutation was present in 1/27 cases of GS where this MT was performed. As a single result such as this should be interpreted with caution, we hope that future studies in this area will be able to better characterize the frequency of this mutation in GS.

Pathological changes in *EGFR*, are typical of the Classical sub-type of GB. We observed fewer pathological changes in *EGFR* in GS than GB, assayed by CNA and NGS. The *EGFRvIII* mutation was also less frequent in GS. In keeping with our findings, pathological alterations in EGFR in GS have already been shown to be relatively infrequent in a series of 15 cases (2/15 with gains at the *EGFR* locus, 2/15 with *EGFR* CNA) [39]. The relationship between *EGFR* and EMT appears to be more complex than simply cause and effect. On the one hand, *EGFR* activation is required for EMT induced by the cytokine TGF-beta1 [40]. However, EMT can be a strategy by which a tumor can escape it’s dependence on *EGFR* activity, e.g. in NSCLC [20, 41]. Thus, we may say that increased *EGFR* activity facilitates EMT and may be involved in the early stages of the process, but is neither necessary nor sufficient for the continued viability of the tumor as EMT progresses. Over-activation of this pathway has been proposed to play a role in EMT, although GB may ‘escape’ from EGFR-targeted therapies [35]. This finding is supported by the experimental work and literature review on this question by Lowder et al., discussed below [42].

SHP-2 (PTPN11) is a key downstream mediator of number of pathways and is activated following receptor activation of NTRK1, EGFR and PD-1 among others, as shown in Fig. 1. *SHP-2* mutations are already recognized in a variety of tumors. These are typically activating. High levels of SHP-2 are recognized as a mediator of EMT, in part through the SHP2-ERK1/2-Snail/Twist1 signaling pathway [14]. *SHP-2* expression has been shown to mediate EMT in a cell culture model of GB which is driven by over-activity of the PDGFRA receptor (Proneural sub-type). PDGFR-alpha amplification is seen particularly in the proneural sub-type of GB, which, as above, is thought to have a particular tendency towards EMT [15, 35]. *SHP-2* mutation has also been highlighted in a case report of recurrence of GB as GS [43].

CALR, a chaperone protein, is found primarily in the endoplasmic reticulum, where it is involved in cell adhesion. It is found as a cell-surface receptor, activating the JAK/MAPK and JAK/STAT pathways. CALR is also seen in the nucleus, where it appears to regulate transcription. High levels of CALR have been shown to adversely affect patient survival in a variety of cancers [44]. CNA of calreticulin (*CALR* or *CRT*), has received relatively little attention as a mediator of EMT. Higher levels of the protein have been identified in a neuroblastoma cell line derived from a bone metastasis displaying EMT [9].

NF1 is a negative regulator of the Ras signal transduction pathway, which is primarily involved in mitosis. As above, loss of function of NF1 is characteristic of the Mesenchymal sub-type of GB. Loss of NF1 has been shown to facilitate EMT in a model of cardiac embryogenesis [16]. A Schwann cell model with this loss of function also showed EMT, which was associated with an increase in reactive oxygen species [45]. These authors suggest that antioxidants may be clinically useful in preventing the progression of such tumors.

NTRK1 is a cell-surface receptor which activates the MAPK pathway. Our finding that *NTRK1* (TrkA) fusion may be associated with GS or with EMT is novel. *NTRK1* fusions have been observed in a small subset of cases of GB [46]. This fusion has been recognized in pediatric mesenchymal tumors, particularly infantile fibrosarcoma and cellular congenital mesoblastic nephroma [47]. Over-expression of TrkB, a member of the same protein family, has been associated with EMT [10]. Identifying tumors with *NTRK1* fusion has become important therapeutically since the FDA-approval of entrectinib as a molecularly targeted treatment for this fusion [48]. As this is a rare occurrence in GB (approximately 1% of cases), performing a randomized trial in this setting to is likely to be challenging [49].

Typical values of the TML vary with the tumor type. Thus, in a panel such as this, which is being used for a variety of tumors, the use of a ‘standard’ cutoff is likely to be less meaningful than a tumor-specific cutoff. The lower value of the optimal cutoff which we found here (8 vs. 17 per MB) is in keeping with the relatively lower TML in GB in comparison with other tumor types. The TML is generally higher in tumors which respond well to immuno-therapy targeting the binding of PD-L1 to PD-1 (atezolizumab, nivolumab etc.). Like PD-L1, TML has been proposed as a bio-marker of response to such therapy [26].

### Comparison to existing literature

A number of case series have used MP to characterize GS. Wojtas et al. examined 10 cases of GS and compared their findings to cases of GB characterized as part of the Cancer Genome Atlas [50]. Six tumors underwent RNA sequencing and gene-expression profiling; as might be expected, 4/6 of the gene-expression profiles were of the Mesenchymal sub-type of GB. Their methods also included NGS with a panel of 664 cancer-related genes (with validation via Sanger sequencing, as indicated). They identified activating mutations in the PI3K/Akt (*PTEN, PI3K*) and RAS/MAPK (*NF1, BRAF*) pathways. They highlighted *PTEN* as the most frequently altered gene in GS, in 7/10 cases, which is higher than the rate of 50% reported for GB in the literature. However, our results with a larger sample size did not confirm alterations in *PTEN* to be more common in GS, as assessed by NGS, CNA and IHC. Nor did we see any significant differences in changes in *PI3K* or *BRAF*.

Cho et al. used compared GB (*n*=90, from a national registry) with GS using, in the latter case, whole-exome sequencing (*n*=28) and CNV (*n*=5) [51]. *TP53* mutations were the most striking difference, being more common in GS (20/28, 70% of cases) vs. GB (29/90, 32%). We were unable to confirm this finding in our series, with no significant difference in the rates of *TP53* mutations in GS vs. GB, assessed via NGS. They also used gene set enrichment analysis to determine if gene sets known to be involved in EMT were more affected in GS. Like Wojtas et al., they found the following pathways to be more frequently altered in GS: RAS/MAPK (*TP53, EGFR, FGFR1, RASGRF2*, PI3K/Akt (*COL5A1, ITGB7, PAK3, PTEN*. They also observed more frequent alterations in phosphatidylinositol/calcium signaling (*CACNA1F/1I, PLCB3/L1, ITPR1/3*. We were unable to confirm alterations in *PAK3* via NGS or in *FGFR1* (via NGS, fusion or CISH (chromogenic in situ hybridization)) in any cases in our data. The other genes above were not part of our MP.

Lowder et al. in their series of 18 cases used a microarray to examine DNA for single nucleotide polymorphisms and CNV, followed by an analysis of the pathways involved in any changes [42]. Copy-number loss was seen in the WNT, NF-kB and CDKN2A pathways. WNT was not assessed in our MP. Although we saw no evidence of copy-number alteration in *NFKB2* in any of our cases nor in *CDKN2A* in any cases of GS (both assessed via CISH) this does not preclude the possibility of other untested alterations in these pathways in our data. The authors also noted CNA in the *HOXA* (particularly H3K27me) and *EGFR* pathways and suggested that the over-expression of *HOXA* genes may account for the mesenchymal phenotype seen in GS. Our study can neither confirm nor refute this hypothesis as CNA was not used as a MT for the *HOXA* genes in out MP.

We acknowledge a number of shortcomings in the design of this study. We cannot be certain that our genetic tests identified mutations within the tumor rather than germline variants as we did not have matched DNA (e.g. from peripheral blood) available for each tumor specimen analyzed. Some cases may have been misdiagnosed. Although there has been little work on the rates of misdiagnosis of GB, we may estimate that up to 5% may be re-classified as lower grade glioma on secondary review [52]. Thus, greater certainty in the diagnosis could have been achieved with a centralized review. We are not aware of any reports of misdiagnosis of GS as GB, as expected given the striking difference in appearance of the sarcomatous element even on standard H&E staining. Also, there was some selection bias as the decision to pursue MP was made individually with each patient as part of their clinical care. We have no way to estimate the number of cases where MP was not performed e.g. where the patient declined such testing or where the testing was deemed inappropriate by their treating physician (e.g. due to the patient’s performance status or co-morbidities). As above, EMT appears to provide the best explanation for the differences we observed in GS vs. GB. If GS is *not* characterized by EMT, it should be possible to show that some of the core features of EMT are *absent* from a substantial proportion of cases of GS. While this has not specifically been investigated to date, measurements of status of histone methylation or acetylation should be able to provide sufficient evidence to refute this hypothesis, if incorrect [28]. Similarly, if EMyeT provides a better explanation than EMT for the changes seen in GS, measurement of cell surface markers typically seen on myeloid lineage cells should be sufficient to confirm this hypothesis [32].

**Fig. 2:**
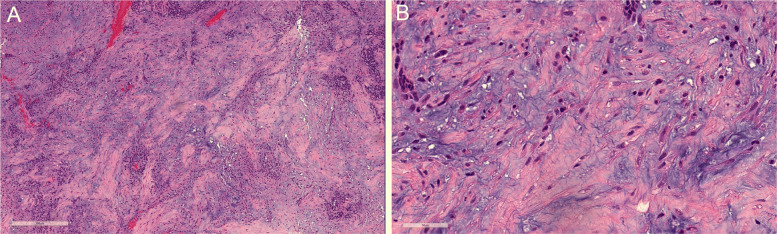
Gliosarcoma without clear glial features (“gliosarcoma *sine* glioblastoma”). The slides show sarcomatous spindle-shaped epithelioid cells and some regions with a chondroid appearance; no clear glial phenotype is evident. Hematoxylin & eosin staining. **A** 85x magnification; scale bar at lower left = 300 microm. **B** 400x magnification; scale bar at lower left = 50 microm

While the WHO definition of GS describes a “biphasic tissue pattern” of glial and sarcomatous elements, we posit that rare cases of GS may appear monophasic, even following excision, with only sarcomatous elements present. This is based on our experience with a number of such cases in at least one of the institutions participating in this study (BNI). A representative case is shown in Fig. 2. Here we see typical spindle-shaped epithelioid cells and some regions with chondroid appearance; no clear glial phenotype is evident. Although this case was not included in our analysis, the tumor was treated as GB and the clinical course confirmed this diagnosis. We may term this phenotype “gliosarcoma *sine* glioblastoma”. Standard H&E staining followed by IHC (typically using stains for GFAP, vimentin, CD34, p53, EGFR and Ki-67) should confirm the diagnosis. Additional IHC may be performed to distinguish cases of sarcoma metastatic to the central nervous system (CNS) and cases of the far rarer primary sarcomas of the CNS. These latter appear to be so uncommon that the relevant literature is comprised of case reports or small case series. These include Kaposi’s sarcoma and lymphomas such as reticulum cell sarcoma as well as primary histocytic sarcoma and primary myeloid sarcoma of the CNS.

## Conclusions

Our MP suggests that, relative to GB, GS exhibits a number of pathological changes, all of which have been associated with EMT. The finding of greater immuno-evasion via PD-1/PD-L1 may become important therapeutically given the number of agents becoming available which target this pathway. Although present only in a single case of GS, the finding of *NTRK1* (TrkA) fusion may also be important in guiding treatment, given the availability of specific inhibitors of this fusion.

The rarity of GS relative to GB underscores the importance of inter-institutional collaboration in characterizing this entity and, in due course, assessing response to treatment. A prospective study with a larger sample size, particularly of GS, using MP designed specifically to investigate GS vs. GB would be helpful in to confirm our findings and to investigate other MTs known to be altered in EMT and possibly in EMyeT.

## Supplementary Information

**Additional file 1** This is a spreadsheet in the Excel file format. It contains the following worksheets:
title-page. The title of the manuscript and the author list.
data1. The data-set used for analysis.
key1. The key to data1 above.
This file can be opened with any common spreadsheet software e.g. LibreOffice, Excel.

**Additional file 2** This is the statistical analysis, provided in.pdf format. This contains the R code used in the analysis and so parts of this are likely to be hard to understand for the reader not familiar with this language. The file provides the results given in the main article. It includes some additional analyses and images which, for reasons of space, do not form part of the main article. In particular, it provides details of *all* of the MTs assessed. For each gene assessed, we also provide hyperlinks (where possible), to the relevant entry at the NCBI Gene database (https://www.ncbi.nlm.nih.gov/gene).

## Data Availability

All data generated or analysed during this study are included in this published article and its supplementary information files.

## Abbreviations

GS: Gliosarcoma
GB: Glioblastoma
MP: Molecular profiling
NGS: Next-generation sequencing
CISH: Chromogenic in situ hybridization
CNV: Copy-number variation
CNA: Copy-number amplification
CNS: Central nervous system
IHC: Immunohistochemistry
FET: Fisher’s exact test
EMT: Epithelial-to-mesenchymal transition
MT: Molecular test
TML: Tumor mutational load
TF: Transcription factor
EMyeT: Epithelial-to-myeloid transition
PDGF: Platelet-derived growth factor
NSCLC: Non–small cell lung cancer
TF: transcription factor
