# Correction to: Gliosarcoma vs. glioblastoma: a retrospective case series using molecular profiling

**DOI:** 10.1186/s12883-021-02326-1

**Published:** 2021-08-14

**Authors:** Christopher Dardis, David Donner, Nader Sanai, Joanne Xiu, Sandeep Mittal, Sharon K. Michelhaugh, Manjari Pandey, Santosh Kesari, Amy B. Heimberger, Zoran Gatalica, Michael W. Korn, Ashley L. Sumrall, Surasak Phuphanich

**Affiliations:** 1 Department of Neurology, Barrow Neurological Institute, Phoenix, AZ USA; 2 School of Medicine, Creighton University, Phoenix, AZ USA; 3 Barrow Brain Tumor Research Center, Department of Neurosurgery, Barrow Neurological Institute, Phoenix, AZ USA; 4 Precision Oncology Alliance, Caris Life Sciences, Phoenix, AZ USA; 5 Fralin Biomedical Research Institute, Virginia Tech Carilion School of Medicine, Roanoke, VA USA; 6 Department of Medical Oncology, West Cancer Center, University of Tennessee Health Science Center, Germantown, TN USA; 7 Pacific Neuroscience Institute and Department of Translational Neurosciences and Neurotherapeutics, John Wayne Cancer Institute, Santa Monica, CA USA; 8 Simpson Querry Biomedical Research Center, Department of Neurosurgery, Feinberg School of Medicine, Northwestern University, Chicago, IL USA; 9 Department of Pathology, University of Oklahoma Health Sciences Center, Oklahoma City, OK USA; 10 Department of Medical Oncology, Levine Cancer Institute, Atrium Health, Charlotte, NC USA; 11 Department of Medicine, Samuel Oschin Comprehensive Cancer Institute, Cedars-Sinai Medical Center, Los Angeles, CA USA

**Correction to: BMC Neurol 21, 231 (2021)**

**https://doi.org/10.1186/s12883-021-02233-5**

Following publication of the original article [1], the authors reported an error that the wrong version of the Additional file 2 was published. The correction version of Additional file 2 is now updated.

The original article [1] has been updated.

## Supplementary Information

**Additional file 2.** This is the statistical analysis, provided in .pdf format. This contains the R code used in the analysis and so parts of this are likely to be hard to understand for the reader not familiar with this language. The file provides the results given in the main article. It includes some additional analyses and images which, for reasons of space, do not form part of the main article. In particular, it provides details of *all *of the MTs assessed. For each gene assessed, we also provide hyperlinks (where possible), to the relevant entry at the NCBI Gene database (https://www.ncbi.nlm.nih.gov/gene).
