# Supplementary material for: Gliosarcoma vs. glioblastoma: a retrospective case series using molecular profiling
Source: BMC Neurol. 2021 Jun 23;21:231. doi: 10.1186/s12883-021-02233-5 (PMC8220715; doi:10.1186/s12883-021-02233-5)
Supplement: Supplementary file 2 — Additional file 2 This is the statistical analysis, provided in.pdf format. This contains the R code used in the analysis and so parts of this are likely to be hard to understand for the reader not familiar with this language. The file provides the results given in the main article. It includes some additional analyses and images which, for reasons of space, do not form part of the main article. In particular, it provides details of all of the MTs assessed. For each gene assessed, we also provide hyperlinks (where possible), to the relevant entry at the NCBI Gene database (https://www.ncbi.nlm.nih.gov/gene). [file 12883_2021_2233_MOESM2_ESM.pdf]

Statistical analysis

A supplement to 'Gliosarcoma vs. Glioblastoma: A retrospective case series using molecular profiling' by Christopher Dardis , David Donner, Nader Sanai, Joanne Xiu, Sandeep Mittal, Sharon K. Michelhaugh, Manjari Pandey, Santosh Kesari, Amy B. Heimberger, Zoran Gatalica, Michael W. Korn, Ashley L. Sumrall and Surasak Phuphanich

Chris Dardis

April 23, 2021

Contents

1 Data **2**
1.1 Defaults for chunks typeset with knitr . . . 2
1.2 Load packages and set options . . . 3
1.3 Load data and key . . . 3
1.4 Sample of data with key . . . 4
1.5 Modify data . . . 6

2 Demographics and other variables **6**
2.1 Demographics . . . 6
2.2 Tumor site . . . 14
2.3 Tumor pathology . . . 20

3 Molecular tests **21**
3.1 Tumor mutational load . . . 21
3.2 Tests with dichotomous outcomes . . . 23
3.3 Methods for tests . . . 24
3.4 Get p values for contingency tables . . . 26
3.5 MT not done . . . 27
3.6 MTs all either +ve or -ve . . . 27
3.7 MTs all -ve . . . 29
3.8 MTs all +ve or -ve in either GB or GS but not both . . . 30
3.9 Summary of MTs . . . 32
3.10 p Values (FET) . . . 33
3.11 MTs with p value of 0.1 to 0.999 . . . 33
3.12 MTs with p value of less than 0.1 . . . 34
3.13 Correlations . . . 35

4 Additional modelling **36**
4.1 Robustness . . . 36
4.2 Recursive partitioning . . . 38
4.3 Logistic regression . . . 42

5 Comparison to existing literature 46

6 R citations 54

7 Data 54

This is an additional file accompanying the main article: Gliosarcoma vs. Glioblastoma: A retrospective case series using molecular profiling.

In the main article, we focus on the molecular tests, which appear first. Here, we proceed in the more traditional manner of first examining variables related to demographics.

Most of the code output/results is in latex format. There are a number of exceptions to this, where the output remains in R format. These code chunks have a slightly different appearance, with a darker background color.

Links within the document appear in dark blue and external hyperlinks appear in pink.

For reasons of space, some output is omitted here. This is marked by `if (interactive()) ...`, indicating that the output will appear when the code is run interactively.

Regarding comments in the code which follows below:

- ### Standard code comments.
- #### Indicates that, for reasons of speed, a result is loaded rather than generated. That is, the code which follows has been commented out and the results it generates are loaded instead. The reader can uncomment this code to generate loaded data themselves.
- ## Code which can be uncommented, if needed, as in the case above. This is also used e.g. to install the required packages, if necessary.

1 Data

Here we load the data and the required packages for analysis.

1.1 Defaults for chunks typeset with knitr

Here we set some parameters to affect how the code chunks are displayed in this document.

```
library('knitr')
### defaults for all chunks
opts_chunk$set(
    eval=TRUE,
    ## text results
    echo=TRUE,
    results=c('markup', 'asis', 'hold', 'hide')[1],
    collapse=FALSE,
    warning=TRUE, message=TRUE, error=TRUE,
    split=FALSE, include=TRUE, strip.white=TRUE,
    ## code decoration
    tidy=FALSE, prompt=FALSE, comment='##',
    highlight=TRUE, size='normalsize',
    background=c('#F7F7F7', colors()[479], c(0.1, 0.2, 0.3))[1],
    ## cache
    cache=FALSE,
    ## plots
    fig.path=c('figure', 'figure/minimal-')[1],
```

```
    fig.keep=c('high', 'none', 'all', 'first', 'last')[1],
    fig.align=c('center', 'left', 'right', 'default')[1],
    fig.show=c('hold', 'asis', 'animate', 'hide')[1],
    dev=c('pdf', 'png', 'tikz')[1],
    fig.width=7, fig.height=7, #inches
    fig.env=c('figure', 'marginfigure')[1],
    fig.pos=c('', 'h', 't', 'b', 'p', 'H')[1])
opts_knit$set(out.format="latex")
knit_theme$set("biogoo")
### Set R options
options(formatR.arrow=TRUE, width=60)
knit_hooks$set(inline = function(x) {
    ## if (is.numeric(x)) return(knitr:::format_sci(x, 'latex'))
    highr::hi_latex(x)
})
```

1.2 Load packages and set options

```
## suppressMessages(source("http://bioconductor.org/biocLite.R"))
## biocLite("reutils")
p1 <- list("gnumeric", "data.table",
           "xtable",
           "ggplot2", "usmap",
           "cutpointr", "reutils",
           "rpart", "rpart.plot",
           "LogisticDx")
## lapply(p1, install.packages, character.only=TRUE)
invisible(lapply(X=p1, FUN=function(x)
    suppressPackageStartupMessages(library(x, character.only=TRUE))))
options("xtable.include.rownames"=FALSE)
options("xtable.booktabs"=TRUE)
### use formatC to print a number with 2 significant digits
fc2 <- function(x, digits=2L) trimws(formatC(x, digits=digits))
```

1.3 Load data and key

The data is loaded from a gnumeric file. This is simpler than loading from Excel. The data is in the same format as the Excel spreadsheet accompanying the main article.

```
### d1 = data
stopifnot(dim(d1 <- data.table(
                  read.gnumeric.sheet("./gb-gs.gnumeric",
                                      head=TRUE,
                                      sheet.name="data1"))) == c(1493, 1184))
### k1 = key
stopifnot(dim(k1 <- data.table(
                  read.gnumeric.sheet("./gb-gs.gnumeric",
                                      head=TRUE,
                                      sheet.name="key1"))) == c(1476, 4))
```

1.4 Sample of data with key

```
print(xtable(k1[c(1:8, 251:304), ],
             align=c("l", "c", "l", "c", "l"),
             caption="A sample of the key to the data. See the accompanying spreadsheet for
             label="tab:key"),
      NA.string="NA",
      tabular.environment="longtable",
      floating=FALSE)
```

column	full.name	values	values.meaning
		NA	not available
		NaN	not a number (not applica
			quality not sufficient
			or result = indeterminate
id	identifier		
deId	deidentified ID		
DEMOGRAPHICS			
year	year pathology sample reported		
HISTOLOGY			
gs	gliosarcoma?	0	no i.e. glioblastoma
		1	yes
lin	lineage	gb	glioblastoma
		lgg	low-grade glioma
rec	recurrent or residual?	0	no
		1	yes
nec	necrosis reported?	0	no
		1	yes
bx	biopsy-only?	0	no
	includes excisional biopsy	1	yes
	but not "excision and biopsy"		
od	oligodendroglial features?	0	no
		1	yes
gc	giant cell?	0	no
		1	yes
hem	intratumoral hemmorhage?	0	no
		1	yes
ep	epithelioid?	0	no
		1	yes
gem	gemistocytic?	0	no
		1	yes
sc	small-cell?	0	no
		1	yes
te	treatment effect?	0	no
		1	yes
fib	fibrillary?	0	no
		1	yes
pOF	pathology - other features		
MOLECULAR TESTS			
tmb	tumor mutational burden		mutations/ MB
	aka tumor mutational load		1 MB = 1 million base pai

	via somatic nonsynonymous missense mutations
htmb	high tumor mutational burden
	i.e. >=17 mutations/MB
msi	microsatellite instability
	via NGS
Key to column name suffixes	
-ci	chromogenic in situ hybridization
-c	copy number amplification
-fa	fragment analysis
-fi	FISH (fluorescence in-situ hybridization)
-fv	fusion variant
-f	fusion via RNA sequencing (ArcherDx Fusionplex)
-ffa	fusion or fragment analysis
-hs	H score (immunohistochemistry score)
-i	immunohistochemistry
-n	next generation sequencing
-ps	pyrosequencing
-r	restriction fragment length polymorphism
-s	Sanger sequencing
List of tests	
cMET	
EGFR	

Table 1: A sample of the key to the data. See the accompanying spreadsheet for the complete key.

```
xtable(head(d1[, .SD, .SDcols=3:18], 15),
       align=c("l", "l", rep("c", 15)),
       caption="A sample of the data; see key (table~\\ref{tab:key}) for abbreviations",
       label="tab:data")
```

year	inst	state	age	ageR	gen	s1	s2	s3	lat	gs	lin	rec	nec	bx	od
2012	jef	pa	57	58	m	l	f	NaN	l	1	gb	0	0	0	0
2012	ucsd	ca	60	62	m	l	f	NaN	r	0	gb	0	0	0	0
2013	scr	ca	59	59	f	l	f	NaN	r	0	gb	0	0	0	0
2013	ucsd	ca	59	59	f	l	t	NaN	l	0	gb	0	0	0	0
2012	irv	ca	50	51	m	ST	ST	NaN		0	gb	0	0	0	0
2010	sjh	az	60	60	m	ST	TH	NaN	c	0	gb	0	0	1	0
2012	jef	pa	67	68	m	l	f	NaN	r	0	gb	0	0	0	0
2013	bay	tx	61	62	f	l	t	NaN	r	0	gb	0	0	1	0
2013	prov	wa	77	77	f	ST	ST	NaN	r	0	gb	0	0	0	0
2013	scr	ca	52	52	m	l	f	NaN	l	0	gb	0	0	0	0
2012	ucsd	ca	52	53	m	ST	TH	NaN	l	0	gb	0	0	0	0
2013	cap	nj	61	61	m	l	t	NaN	r	0	gb	0	0	0	0
2013	cap	nj	47	47	m	l	f	NaN	r	0	gb	0	0	0	0
2013	cap	nj	38	38	m	l	p	NaN	l	0	gb	0	0	0	0
2013	cap	nj	48	48	f	l	f	NaN	r	0	gb	0	0	0	0

Table 2: A sample of the data; see key (table 1) for abbreviations

1.5 Modify data

```
cat("\n \\quote{Mismatch repair status is determined by the presence or absence of the repa
```

Mismatch repair status is determined by the presence or absence of the repair proteins MLH1, MSH2, MSH6 and PMS2 by IHC. If any of these IHC's are negative, mismatch repair status is considered deficient.

```
### convert these to indicate that when the test is +ve,
###  a pathological change in present, as
###  this is how all of the other tests are reported
### mrs1 = mismatch repair status
mrs1 <- c("MLH1.i", "MSH2.i", "MSH6.i", "PMS2.i")
for (i in seq_along(mrs1)) {
    ## print(d1[, sum(is.finite(unlist(.SD))), .SDcols=n1[i]])
    set(d1, j=mrs1[i], value=d1[, as.numeric(!.SD), .SDcols=mrs1[i]])
}
### GS as factor; we drop this again later
set(d1, j="gsf", value=factor(d1$gs, levels=c(0, 1),
                              labels=c("GB", "GS")))
### remove cases of GB which were Bx only
t1 <- d1[, table(
    factor(bx, levels=c(0, 1), labels=c("excision", "biopsy only")),
    gsf)]
print(xtable(t1,
             caption="Biopsy only X GB vs. GS"),
      include.rownames=TRUE)
```

	GB	GS
excision	1181	42
biopsy only	264	6

Table 3: Biopsy only X GB vs. GS

```
d1 <- d1[!(d1$gs==0 & d1$bx==1), ]
```

2 Demographics and other variables

2.1 Demographics

```
cat(paste0("Cases which are GS: ",
           d1[, sum(gs)], " of ", d1[, sum(is.finite(gs))], " = ",
           fc2(d1[, sum(gs)] / d1[, sum(is.finite(gs))] * 100),
           " \\%"))
```

Cases which are GS: 48 of 1229 = 3.9 %

```
cat(paste0("Proportion of cases with information on year of sample = ",
           fc2(d1[, sum(is.finite(year))] / nrow(d1) * 100, digits=3),
           " \\%"))
```

Proportion of cases with information on year of sample = 20.4 %

```
xtable(d1[, list("year"=names(summary(factor(year))),
                 "n"=summary(factor(year)))],
       align=c("l", "l", "c"),
       caption="Year X number of cases")
```

year	n
2009	2
2010	3
2011	7
2012	32
2013	117
2014	90
NA's	978

Table 4: Year X number of cases

```
invisible(d1[, barplot(table(factor(year)),
                       ylim=c(0, 140), col="grey50",
                       xlab="Year", ylab="Number of cases",
                       main="Year X number of cases",
                       cex.names=1.6, cex.main=2,
                       cex.lab=1.4, cex.axis=1.2)])
t1 <- d1[, table(year, gsf)]
### using simulate.p.value for tables with cells which have zero values
###  values for p may vary by +- 0.02 (aprox.)
###  using set.seed to make results reproducible
set.seed(seed=1)
p1 <- fc2(chisq.test(t1, simulate.p.value=TRUE)$p.value)
print(xtable(t1,
             caption=paste0("Year X GB vs. GS; chi-square: p=", p1)),
      include.rownames=TRUE)
```

	GB	GS
2009	2	0
2010	3	0
2011	7	0
2012	30	2
2013	114	3
2014	89	1

Table 5: Year X GB vs. GS; chi-square: p=0.5

```
#### institution
### The geographic location of each case is given according to that of
###  the corresponding Pathology Laboratory/Department.
### We acknowledge that there may have been cases where surgical samples
###  from additional, remote institutions were processed at one Laboratory,
###  although this would have been an insignificant minority, if any.
set(d1, j="inst", value=factor(d1$inst))
set(d1, j="state", value=factor(d1$state))
cat(paste0("Proportion of cases with information on institution = ",
           fc2(d1[, sum(is.finite(inst))] / nrow(d1) * 100, digits=3),
           " \\%"))
```

Proportion of cases with information on institution = 19.4 %

```
### using -1 below to remove those where inst == NA
r1 <- d1[, sort(summary(inst), decreasing=TRUE)]
r2 <- r1[-1]
paste0("Number of institutions = ",
       n1 <- length(r2))
```

[1] "Number of institutions = 79"

```
paste0("Number of institutions with at least one case = ",
       n1gr1 <- length(r2[r2 > 1]))
```

[1] "Number of institutions with at least one case = 31"

```
paste0("Proportion of institutions with at least one case = ",
       fc2(n1gr1 / n1 *100),
       " \\%")
```

[1] "Proportion of institutions with at least one case = 39

```
r2 <- vector(mode="character", length=length(r1))
suppressMessages(
    for (i in 2:length(r1))
        r2[i] <- d1[d1$inst==names(r1)[i],
                    toupper(as.character(first(state)))]
)
r2 <- cbind(c(names(r1), NA), c(r2, NA), c(r1, NA))
l1 <- list()
for (i in seq.int(length.out=9)){
    s1 <- seq(from=(i * 9) - 8, to=i * 9)
    l1[[i]] <- data.frame(matrix(data=c(r2[s1, ]), nrow=3, byrow=TRUE))
}
r2 <- rbindlist(l1)
set(r2, j="rownames", value=rep(c("institution", "state", "n"), times=9))
setcolorder(r2, neworder=c(10, seq(9)))
print(
    xtable(r2,
```

```
          caption="Institutions (abbreviated), with state and number of cases. See Key
          align=c(rep("l", 2), rep("c", 9))),
    include.colnames=FALSE)
```

institution	NA's	jef	meth	luke	fh	ucsd	penn	cap	scr
state		PA	TX	TX	FL	CA	PA	NJ	CA
n	991	31	26	18	12	12	11	9	9
institution	sjh	scot	ced	caro	froed	harp	kar	sam	arl
state	AZ	AZ	CA	NC	WI	MI	MI	NY	TX
n	8	5	4	3	3	3	3	3	2
institution	bay	chi	fhmc	gros	gt	hous	mskcc	msl	nit
state	TX	AR	FL	CA	DC	TX	NY	TX	PA
n	2	2	2	2	2	2	2	2	2
institution	plano	prov	sharp	sinai	tex	ana	apmc	bhl	cam
state	TX	WA	CA	FL	TX	CA	LA	KY	CA
n	2	2	2	2	2	1	1	1	1
institution	cc	cfpa	cop	cp	cyp	eis	emc	falls	fran
state	OH	FL	OR	AZ	TX	CA	PA	ID	
n	1	1	1	1	1	1	1	1	1
institution	geis	gulf	holy	imm	irv	lamc	lan	lima	mort
state	PA	FL	PA	NE	CA	CA	PA	OH	FL
n	1	1	1	1	1	1	1	1	1
institution	msj	muir	ns	nw	nyu	oak	phmc	pin	river
state	WI	CA	NY	IL	NY	MI	MI	PA	CA
n	1	1	1	1	1	1	1	1	1
institution	rml	sb	shand	shore	sjr	spec	stan	sv	theda
state	OK	CA	FL	MD	NJ	MI	CA	CA	WI
n	1	1	1	1	1	1	1	1	1
institution	ucsf	upmc	vand	vv	wash	west	wjef	yale	
state	CA	PA	TN	CO	WA	NY	LA	CT	
n	1	1	1	1	1	1	1	1	

Table 6: Institutions (abbreviated), with state and number of cases. See Key (table 1) for abbreviations

```
### institution x GS
t1 <- d1[, table(inst, gsf, useNA="ifany")]
p1 <- fc2(chisq.test(t1, simulate.p.value=TRUE)$p.value)
m1 <- as.matrix(t1[rowSums(t1) > 1, ])
m1 <- m1[order(rowSums(m1), decreasing=TRUE), ]
p1 <- fc2(chisq.test(m1[-1, ], simulate.p.value=TRUE)$p.value)
print(xtable(m1[-1, ], align=c("l", "c", "c"),
             caption=paste0("Institution (removing institutions with just one case and
      tabular.environment="longtable",
      floating=FALSE,
      include.rownames=TRUE)
```

	GB	GS
jef	30	1
meth	26	0
luke	18	0

fh	12	0
ucsd	12	0
penn	11	0
cap	8	1
scr	8	1
sjh	8	0
scot	5	0
ced	4	0
caro	2	1
froed	3	0
harp	3	0
kar	3	0
sam	3	0
arl	2	0
bay	2	0
chi	2	0
fhmc	2	0
gros	2	0
gt	2	0
hous	2	0
mskcc	2	0
msl	2	0
nit	2	0
plano	2	0
prov	2	0
sharp	2	0
sinai	2	0
tex	2	0

Table 7: Institution (removing institutions with just one case and NAs) X pathology (GB vs. GS), ; p (chi-square)= 0.59

```
### state
cat(paste0("Proportion of cases with information on state = ",
           fc2(d1[, sum(is.finite(state))] / nrow(d1) * 100, digits=3),
           " \\%"))
```

Proportion of cases with information on state = 19.3 %

```
r1 <- d1[, sort(summary(state), decreasing=TRUE)]
r2 <- r1[-1]
paste0("Number states = ",
       n1 <- length(r2))
```

[1] "Number states = 25"

```
paste0("Number states with at least one case = ",
       n1gr1 <- length(r2[r2 > 1]))
```

[1] "Number states with at least one case = 14"

```
cat(paste0("Proportion of states with at least one case = ",
           fc2(n1gr1 / n1 *100),
           " \\%"))
```

Proportion of states with at least one case = 56 %

```
s1 <- d1[, summary(state)]
s1 <- data.frame("n"=s1, "state"=names(s1))
usmap::plot_usmap(data=s1, values="n", labels=TRUE) +
    scale_fill_continuous(type="viridis",  na.value="grey90",
                          name="Number\nof cases", label=scales::comma) +
    labs(title="Number of cases, by state",
            subtitle="States filled in grey have zero cases") +
    theme(legend.position="right",
          legend.title=element_text(size=18),
          legend.text=element_text(size=16),
          plot.title=element_text(size=20, hjust=0.5),
          plot.subtitle=element_text(size=16, hjust=0.5))
### institution x GS
t1 <- d1[, table(state, gsf, useNA="ifany")]
p1 <- fc2(chisq.test(t1, simulate.p.value=TRUE)$p.value)
m1 <- as.matrix(t1[rowSums(t1) > 1, ])
m1 <- m1[order(rowSums(m1), decreasing=TRUE), ]
dimnames(m1)$state  <- toupper(dimnames(m1)$state)
p1 <- fc2(chisq.test(m1[-1, ], simulate.p.value=TRUE)$p.value)
print(xtable(m1[-1, ], align=c("l", "c", "c"),
             caption=paste0("State (removing states with just one case and NAs) X patho
      include.rownames=TRUE)
```

	GB	GS
TX	56	0
PA	50	2
CA	36	2
FL	20	0
AZ	14	0
NJ	9	1
NY	10	0
MI	9	0
WI	5	0
NC	2	1
WA	3	0
DC	2	0
LA	2	0
OH	2	0

Table 8: State (removing states with just one case and NAs) X pathology (GB vs. GS); p (chi-square)=0.25

```
### age
### there are two values for age
####  'age' (at collection date) is not significantly different from
```

```
####  'ageR' (age reported)
## d1[, t.test(age, ageR, alternative="less")]
r1 <- rbindlist(list(
    c("GB", d1[gs==0, as.list(c(summary(age), "n"=sum(is.finite(age))))]),
    c("GS", d1[gs==1, as.list(c(summary(age), "n"=sum(is.finite(age))))])))
p1 <- fc2(t.test(d1[gs==0, age], d1[gs==1, age])$p.value)
p2 <- fc2(t.test(d1[gs==0, age], d1[gs==1, age],
                alternative="greater")$p.value)
xtable(r1, digits=c(0, 0, 0, rep(1, 4), 0, 0),
       caption=paste0("Age X pathology (GB vs. GS), t-test: equal, p=", p1,
         ", GS younger: p=", p2))
```

V1	Min.	1st Qu.	Median	Mean	3rd Qu.	Max.	n
GB	4	49.0	58.0	56.7	66.0	90	1181
GS	26	48.0	55.0	53.8	60.2	78	48

Table 9: Age X pathology (GB vs. GS), t-test: equal, p=0.084, GS younger: p=0.042

```
### gender
r1 <- rbindlist(list(
    c("GB", d1[gs==0, as.list(c(summary(gen), "n"=sum(is.finite(gen))))]),
    c("GS", d1[gs==1, as.list(c(summary(gen), "n"=sum(is.finite(gen))))])))
p1 <- fc2(fisher.test(t(r1[, 2:3]), alternative="two.sided")$p.value)
### Proportion of F lower in GS vs. GB i.e. more M affected in GB
p2 <- fc2(fisher.test(t(r1[, 2:3]), alternative="less")$p.value, digits=3)
xtable(r1,
       caption=paste0("Gender X pathology (GB vs. GS). FET (Fisher's exact test): equal
```

V1	f	m	n
GB	448	733	1181
GS	23	25	48

Table 10: Gender X pathology (GB vs. GS). FET (Fisher's exact test): equal, p=0.17, more GB in M: p=0.108

```
### as proportions
r2 <- d1[, fc2(prop.table(table(gen, gsf), margin=2))]
print(xtable(r2,
             caption="Gender X pathology (GB vs. GS). Proportion of females/males per t
      include.rownames=TRUE)
```

	GB	GS
f	0.38	0.48
m	0.62	0.52

Table 11: Gender X pathology (GB vs. GS). Proportion of females/males per tumor type

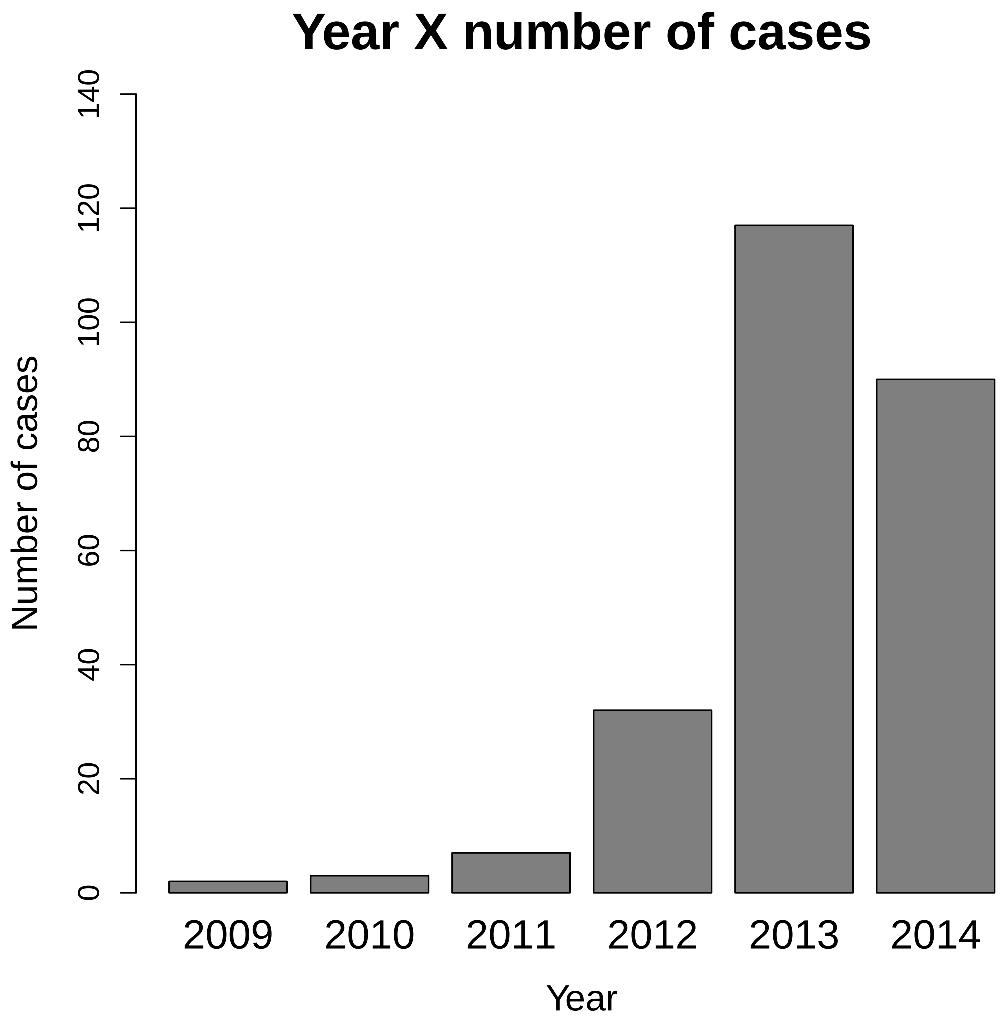
Year X number of cases
Number of cases
0
20
40
60
80
100
120
140
2009
2010
2011
2012
2013
2014
Year

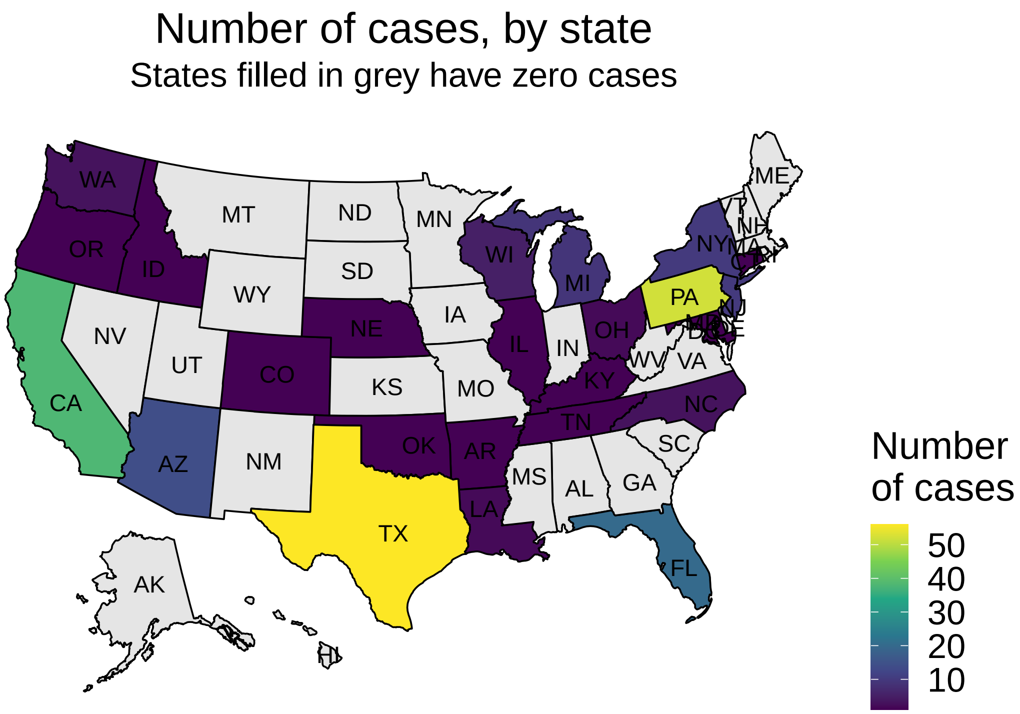

2.2 Tumor site

```
### s1 = short/ brief description of tumor site
cat(paste0("\n Proportion of cases with information on site = ",
       fc2(d1[, sum(is.finite(s1))] / nrow(d1) * 100, digits=3),
       " \\%"))
```

Proportion of cases with information on site = 100 %

```
xtable(k1[seq.int(from=which(k1$column=="s1"),
                  to=which(k1$column=="s2") - 1),
          list(values, "meaning"=values.meaning)],
       align=c("l", "l", "c"),
       caption="Key to site (short)")
```

values	meaning
B	brain
l	lobar
ml	multi-lobar
	i.e. >= 2 lobes
ST	supra-tentorial
IT	infra-tentorial
SC	spinal cord
NNS	non nervous-system

Table 12: Key to site (short)

```
r1 <- rbindlist(list(
  c("GB", d1[gs==0, as.list(summary(s1))]),
  c("GS", d1[gs==1, as.list(summary(s1))])))
setcolorder(r1, neworder=c(1, 4, 2, 5, 8))
p1 <- fc2(chisq.test(t(r1[, 2:3]), simulate.p.value=TRUE)$p.value)
xtable(r1,
       caption=paste0("Pathology (GB vs. GS) X Site (short) X ; p (chi-square)=", p1),
       label="tab:s1")
```

V1	l	B	ml	ST	IT	NNS	SC
GB	877	171	82	33	11	3	4
GS	37	7	3	0	0	1	0

Table 13: Pathology (GB vs. GS) X Site (short) X ; p (chi-square)=1

```
### supra/infra-tentorial
set(d1, j="s1st",
    value=d1[, factor(s1, levels=levels(s1),
                      labels=c("st", "it", "st", "st", NaN, NaN, "st"))])
r1 <- rbindlist(list(
  c("GB", d1[gs==0, as.list(summary(s1st))]),
  c("GS", d1[gs==1, as.list(summary(s1st))])))
setnames(r1, old=4, new="NA")
p1 <- fc2(chisq.test(r1[, 2:4], simulate.p.value=TRUE)$p.value)
p2 <- fc2(fisher.test(r1[, 2:3], alternative="less")$p.value)
xtable(r1,
       caption=paste0("Pathology (GB vs. GS) X Site (supra- vs. infra-tentorial); n=",
"; excluding NaNs, p=", p2))
```

V1	st	it	NA
GB	1163	11	7
GS	47	0	1

Table 14: Pathology (GB vs. GS) X Site (supra- vs. infra-tentorial); n=1221; all cases, p=0.33; excluding NaNs, p=0.65

```
### brain vs. spinal cord
set(d1, j="s1sc",
    value=d1[, factor(s1, levels=levels(s1),
                      labels=c("b", "b", "b", "b", "NaN", "sc", "b"))])
r1 <- rbindlist(list(
  c("GB", d1[gs==0, as.list(summary(s1sc))]),
  c("GS", d1[gs==1, as.list(summary(s1sc))])))
setcolorder(r1, neworder=c(1, 2, 4))
p1 <- fc2(chisq.test(r1[, 2:4], simulate.p.value=TRUE)$p.value)
p2 <- fc2(fisher.test(r1[, 2:3], alternative="less")$p.value)
xtable(r1,
       caption=paste0("Pathology (GB vs. GS) X Site (brain vs. spinal cord), n=", sum(r
                      "; excluding NAs and NaNs, p=", p2))
```

V1	b	sc	NaN
GB	1174	4	3
GS	47	0	1

Table 15: Pathology (GB vs. GS) X Site (brain vs. spinal cord), n=1225; all cases, p=0.27; excluding NAs and NaNs, p=0.85

```
### brain: single vs. multi-lobar
t1 <- d1[!d1$s1=="NNS", table(s1=="B", gsf)]
p1 <- fc2(fisher.test(t1, alternative="less")$p.value)
print(xtable(t1,
             caption=paste0("Site=brain X Pathology (GB vs. GS); FET p=",
                            p1)),
      include.rownames=TRUE)
```

	GB	GS
FALSE	1007	40
TRUE	171	7

Table 16: Site=brain X Pathology (GB vs. GS); FET p=0.63

```
### metastatic
m1 <- d1[!s3=="NaN", table(s2, s3)]
m1 <- m1[rowSums(m1) >= 1, c("idem", "LN", "rt")]
print(xtable(m1,
             caption="Site; metastatic",
             label="tab:met"),
      include.rownames=TRUE)
```

```
k2 <- k1[c(which(k1$values %in% dimnames(m1)$s2),
           which(k1$values %in% dimnames(m1)$s3)),
         list(values, values.meaning)]
xtable(k2, caption="Key to table~\\ref{tab:met}")
```

	idem	LN	rt
C	1	0	0
N	0	1	0
subCut	0	0	1

Table 17: Site; metastatic

values	values.meaning
C	cervial
N	neck
subCut	subcutaneous
LN	lymph node
idem	intradural, extramedullary
rt	right temporal, subcutaneous tissue

Table 18: Key to table 17

```
xtable(k1[seq.int(from=which(k1$column=="s1"),
                  to=which(k1$column=="s2") - 1),
          list(values, "meaning"=values.meaning)],
       align=c("l", "l", "c"),
       caption=paste0("Site; key to table \\ref{tab:met}, p = ", p1))
```

values	meaning
B	brain
l	lobar
ml	multi-lobar
	i.e. >= 2 lobes
ST	supra-tentorial
IT	infra-tentorial
SC	spinal cord
NNS	non nervous-system

Table 19: Site; key to table 17, p = 0.63

```
### s2 = detailed description
k2 <- k1[seq.int(from=which(k1$column=="s2"),
                 to=which(k1$column=="s3")-1),
         list(values, values.meaning)]
k2 <- cbind(k2[1:(nrow(k2)/2), ],
            k2[((nrow(k2)/2)+1):nrow(k2), ])
print(xtable(k2,
             caption="Key to site (detailed)"),
      tabular.environment="longtable",
      floating=FALSE)
```

values	values.meaning	values	values.meaning
ST	supra-tentorial	THP	thalamus and parietal lobe

CH	cerebral hemisphere	THMB	thalamus and midbrain
	includes:	TH	thalamus
	cerebrum	BG	basal ganglia
	cerebral cortex	BGT	basal ganglia and temporal
f	frontal	IT	infra-tentorial
t	temporal		includes:
ft	fronto-temporal		posterior fossa
p	parietal	BS	brainstem
fp	fronto-parietal	4V	4th ventricle
tp	tempero-parietal	CB	cerebellum
ftp	fronto-tempero-parietal	B	brain
	includes Sylvian fissure	IV	intra-ventricular
o	occipital	PV	peri-ventricular
to	tempero-occipital	SC	spinal cord
po	parieto-occipital	C	cervial
tpo	tempero-parieto-occipital	L	lumbar
DWM	deep white matter	T	thoracic
sCort	subcortical	N	neck
PIN	pineal	PL	pleura
CC	corpus callosum	subCut	subcutaneous

Table 20: Key to site (detailed)

```
k2[, sum(!values=="")]
```

[1] 17

```
### detailed location - table
set(d1, j="s2", value=factor(d1$s2))
r1 <- rbindlist(list(
  d1[gs==0, as.list(summary(s2))],
  d1[gs==1, as.list(summary(s2))]))
p1 <- fc2(d1[, chisq.test(table(gs, s2),
                         simulate.p.value=TRUE)]$p.value)
### transpose for printing
r1 <- t(r1)
dimnames(r1)[[2]] <- c("GB", "GS")
r1 <- r1[order(r1[, "GB"], decreasing=TRUE), ]
print(xtable(r1,
             caption=paste0("Site (detailed) vs. GS; chi-square p=", p1)),
      include.rownames=TRUE,
      tabular.environment="longtable",
      floating=FALSE)
```

	GB	GS
f	327	14
t	322	13
p	176	8
B	168	7
o	51	2

tp	21	2
po	19	0
ft	18	0
fp	16	1
CH	14	0
CB	8	0
to	7	0
TH	5	0
ST	4	0
BG	3	0
CC	3	0
IT	3	0
ftp	2	0
IV	2	0
BGT	1	0
C	1	0
DWM	1	0
L	1	0
N	1	0
PIN	1	0
PL	1	0
SC	1	0
subCut	1	1
T	1	0
THP	1	0
tpo	1	0

Table 21: Site (detailed) vs. GS; chi-square p=0.7

```
## xtable(as.data.frame(d1[, levels(s3)]))
### laterality
t1 <- d1[, table(gsf, lat)]
p1 <- fc2(chisq.test(t1, simulate.p.value=TRUE)$p.value)
### left vs. right - not significant
p2 <- fc2(fisher.test(t1[, 3:4], alternative="less")$p.value)
### central/bilateral vs. unilateral - not significant
t2 <- rbindlist(list(
    as.list(rowSums(t1[,1:2])), as.list(rowSums(t1[,3:4]))))
p3 <- fc2(fisher.test(t2, alternative="greater")$p.value)
print(xtable(k1[seq.int(from=which(k1$column=="lat"),
                        to=which(k1$column=="gs")-1),
                list(values, values.meaning)],
             caption="Laterality; key to table \\ref{tab:lat}"),
      NA.string="NA")
```

```
options("xtable.include.rownames"=TRUE)
xtable(t1, caption=paste0("Pathology (GB vs. GS) X Laterality; chi-square p=", p1),
       label="tab:lat")
```

values	values.meaning
l	left
r	right
b	bilateral
c	central
NA	not available

Table 22: Laterality; key to table 23

	b	c	l	r
GB	5	7	446	518
GS	0	0	20	17

Table 23: Pathology (GB vs. GS) X Laterality; chi-square p=0.61

2.3 Tumor pathology

This details characteristics seen on light microscopy and related variables.

```
t1 <- d1[, table(gs, lin)]
dimnames(t1) <- list(c("GB", "GS"),
                     c("GB", "LGG"))
p1 <- fc2(
    chisq.test(t1,
               simulate.p.value=TRUE)$p.value)
print(xtable(t1,
             caption=paste0(
                 "Pathology (GB vs. GS) X lineage (GB vs. LGG);\n",
                 "chi-square, two-sided, p=", p1)))
```

	GB	LGG
GB	1181	0
GS	47	1

Table 24: Pathology (GB vs. GS) X lineage (GB vs. LGG); chi-square, two-sided, p=0.041

```
(n1 <- colnames(d1)[15:25])
```

[1] "rec" "nec" "bx" "od" "hem" "gc" "ep" "gem" "sc" [10] "te" "fib"

```
names(n1) <- n1
colnames1=c("feature", "p (FET)",
            "nGS", "pGS", "nGB", "pGB",
            "OR")
dt1 <- data.table(matrix(data="",
                         nrow=length(n1), ncol=length(colnames1)))
setnames(dt1, old=seq.int(length(colnames1)), new=colnames1)
for (i in seq.int(n1)) {
```

```
    set(dt1, i=i, j="feature",
        value=as.character(k1[k1$column==n1[i], ]$"full.name"))
    ## ct1 = contingency table
    ct1 <- d1[, table(gsf, get(n1[i]))]
    set(dt1, i=i, j="nGS",
        value=paste(ct1["GS", "1"], "/", sum(ct1["GS", ])))
    set(dt1, i=i, j="nGB",
        value=paste(ct1["GB", "1"], "/", sum(ct1["GB",])))
    set(dt1, i=i, j="pGS",
        value=fc2(100 * ct1["GS", "1"] / sum(ct1["GS", ])))
    set(dt1, i=i, j="pGB",
        value=fc2(100 * ct1["GB", "1"] / sum(ct1["GB", ])))
    set(dt1, i=i, j="p (FET)",
        value=fc2(chisq.test(ct1,
                             simulate.p.value=TRUE)$p.value))
}
set(dt1, j="OR", value=dt1[, fc2(as.numeric(pGS) / as.numeric(pGB))])
dt1 <- dt1[order(as.numeric(OR), decreasing=TRUE), ]
### remove biopsy only as we already dropped cases
###  of GB which were biopsy only
xtable(dt1[!dt1$feature=="biopsy-only?", ],
       caption="Pathological features; sorted by OR",
       align=c("l", "l", rep("c", times=length(colnames1) - 1)),
       label="tab:path")
```

	feature	p (FET)	nGS	pGS	nGB	pGB	OR
1	necrosis reported?	0.63	2 / 48	4.2	31 / 1181	2.6	1.6
2	recurrent or residual?	0.48	7 / 48	15	132 / 1181	11	1.4
3	oligodendroglial features?	0.65	0 / 48	0	18 / 1181	1.5	0
4	intratumoral hemmorhage?	1	0 / 48	0	7 / 1181	0.59	0
5	giant cell?	1	0 / 48	0	7 / 1181	0.59	0
6	epithelioid?	1	0 / 48	0	6 / 1181	0.51	0
7	gemistocytic?	1	0 / 48	0	4 / 1181	0.34	0
8	small-cell?	1	0 / 48	0	6 / 1181	0.51	0
9	treatment effect?	0.67	0 / 48	0	14 / 1181	1.2	0
10	fibrillary?	1	0 / 48	0	1 / 1181	0.085	0

Table 25: Pathological features; sorted by OR

3 Molecular tests

Most of the molecular tests (MTs) given below appear with hyperlinks to the relevant entry in the NCBI's Gene database; these hyperlinks appear in pink.

3.1 Tumor mutational load

```
suppressWarnings(
    set(d1, j="tmb", value=as.numeric(as.character(d1$tmb))))
r1 <- rbindlist(list(
  c("GB", d1[gs==0, as.list(summary(tmb))]),
  c("GS", d1[gs==1, as.list(summary(tmb))])))
setcolorder(r1, neworder=c(1, 2, 4))
p1 <- fc2(t.test(d1[gs==0, tmb], d1[gs==1, tmb])$p.value)
p2 <- fc2(t.test(d1[gs==1, tmb], d1[gs==0, tmb],
                 alternative="greater")$p.value)
xtable(r1,
       caption=paste0("Pathology (GB vs. GS) X tumor mutational load;
t-test: two-sided, p=", p1, "; t-test, GS greater, p=", p2, collpase=""))
```

	V1	Min.	Median	1st Qu.	Mean	3rd Qu.	Max.	NA's
1	GB	1.00	6.00	4.00	8.08	8.00	264.00	560.00
2	GS	1.00	6.00	4.00	12.26	8.50	91.00	21.00

Table 26: Pathology (GB vs. GS) X tumor mutational load; t-test: two-sided, p=0.35; t-test, GS greater, p=0.17

```
### high TMB is TMB > 17 / 10^6 BPs
###  using is.finite to drop NaN from factor levels
t1 <- d1[is.finite(d1$htmb),
         table(gsf, factor(htmb, labels=c("no", "yes")))]
p1 <- fc2(fisher.test(t1, alternative="greater")$p.value)
xtable(t1,
       caption=paste0("Pathology (GB vs. GS) X TMB > 17/10e6 BPs; t-test: GS greater, 
```

	no	yes
GB	600	21
GS	25	2

Table 27: Pathology (GB vs. GS) X TMB > 17/10e6 BPs; t-test: GS greater, p=0.25

```
### check if better cut-off
cut1 <- cutpointr(d1, x="tmb", class="gs", na.rm=TRUE,
                  pos_class=1, direction=">=")$optimal_cutpoint
t1 <- d1[is.finite(d1$tmb),
         table(gsf, factor(as.numeric(tmb) >= cut1, labels=c("no", "yes")))]
p1 <- fc2(fisher.test(t1, alternative="greater")$p.value)
xtable(t1,
       caption=paste0("Pathology (GB vs. GS) X TMB > ", cut1, "/1,000,000 BPs; t-test:
```

```
### remove newly added columns above
set(d1, j=c("gsf", "s1sc", "s1st"), value=NULL)
```

	no	yes
GB	454	167
GS	16	11

Table 28: Pathology (GB vs. GS) X TMB > 8/1,000,000 BPs; t-test: GS greater, p=0.09

3.2 Tests with dichotomous outcomes

These tests are all binary i.e. the result is either positve or negative.

```
### molecular tests, binary
if (interactive()) names(d1)
length(mt1 <- names(d1)[c(29:length(d1))])
```

[1] 1156

```
### check if any were all NAs
na1 <- d1[, sapply(.SD, FUN=function(x) all(is.na(x))), .SDcols=mt1]
na1 <- names(na1[which(na1)])
xtable(data.table(na1),
       caption="All results=NA (not availble) for these tests")
```

	na1
1	EGFR_T790M.c
2	H3K36me3.i
3	EGFR_T790M.n
4	EGFR_Tertiary_Mutation.n

Table 29: All results=NA (not availble) for these tests

```
### drop them
set(d1, j=na1, value=NULL)
length(mt1 <- names(d1)[c(29:length(d1))])
```

[1] 1152

```
### number of tests done for each case
set(d1, j="ntests",
    value=d1[, sum(is.finite(unlist(.SD))), .SDcols=mt1, by=id][, V1])
r1 <- rbindlist(list(
    c("all", d1[, as.list(summary(ntests))]),
    c("GB", d1[gs==0, as.list(summary(ntests))]),
    c("GS", d1[gs==1, as.list(summary(ntests))])))
## p1 <- fc2(t.test(d1[gs==0, ntests], d1[gs==1, ntests])$p.value)
p2 <- fc2(t.test(d1[gs==0, ntests], d1[gs==1, ntests],
                 alternative="less")$p.value)
xtable(r1,
       caption=paste0("Pathology (GB vs. GS) X number of tests; t-test, fewer in GS vs
```

	V1	Min.	1st Qu.	Median	Mean	3rd Qu.	Max.
1	all	0.00	63.00	946.00	564.78	1031.00	1092.00
2	GB	0.00	63.00	946.00	564.07	1031.00	1092.00
3	GS	11.00	70.00	823.50	582.35	1030.25	1073.00

Table 30: Pathology (GB vs. GS) X number of tests; t-test, fewer in GS vs. GB, p=0.4

```
### year
l1 <- list()
for (i in seq.int(from=min(d1$year, na.rm=TRUE),
                  to=max(d1$year, na.rm=TRUE))){
    l1[[as.character(i)]]  <-
        d1[year==i, as.list(c("year"=i, "n"=.N, summary(ntests)))]}
xtable(rbindlist(l1),
       caption="Year vs. summary of number of molecular tests",
       digits=0)
```

	year	n	Min.	1st Qu.	Median	Mean	3rd Qu.	Max.
1	2009	2	59	60	62	62	64	65
2	2010	3	9	28	46	39	54	61
3	2011	7	36	49	53	54	62	66
4	2012	32	6	56	61	55	63	70
5	2013	117	0	54	62	53	64	70
6	2014	90	1	60	66	57	70	72

Table 31: Year vs. summary of number of molecular tests

```
set(d1, j="ntests", value=NULL)
```

3.3 Methods for tests

```
d1[, all(is.na(MSI.fa))]
```

[1] FALSE

```
### type of test
r1 <- regmatches(mt1, regexpr(pattern="[^.]+$",  text=mt1))
dt1 <- data.table(t(sort(table(r1), decreasing=TRUE)))
set(dt1, j="V2", value=NULL)
setnames(dt1, new=c("abbreviation", "n"))
set(dt1, j="test",
    value=c("next generation sequencing",
            "copy number amplification",
            "fusion via RNA sequencing",
            "immunohistochemistry",
            "Sanger sequencing",
            "FISH (fluorescence in-situ hybridization)",
            "chromogenic in situ hybridization",
```

```
              "restriction fragment length polymorphism",
              "fragment analysis",
              "fusion variant",
              "fusion or fragment analysis",
              "H score (immunohistochemistry score)",
              "microsatellite instability",
              "pyrosequencing"))
setcolorder(dt1, neworder=c(1, 3, 2))
## < 100 char, 50x left
for (i in seq.int(nrow(dt1))){
    p1 <- paste("[.]", dt1[i, abbreviation] , "$", sep="")
    m1 <- mt1[grepl(p1, mt1)][1:5]
    m1 <- m1[which(!is.na(m1))]
    set(dt1, i=i, j="examples",
        value=paste0(m1, collapse=", "))
}
xtable(dt1,
       align=c("c", "c", "l", "c", "l"),
       caption="Method for molecular tests, sorted by number of tests")
```

	abbreviation	test	n	examples
1	n	next generation sequencing	597	X43348.n, X43349.n, X43352.n, ABI1.n, ABL1.n
2	c	copy number amplification	443	X43352.c, ABL2.c, ACSL3.c, ACSL6.c, ADGRA2.c
3	f	fusion via RNA sequencing	54	AKT3.f, ALK.f, ARHGAP26.f, AXL.f, BRAF.f
4	i	immunohistochemistry	27	ALK.i, Androgen_Receptor.i, cMET.i, EGFR.i, ER.
5	s	Sanger sequencing	9	BRAF.s, BRCA1.s, BRCA2.s, c.KIT.s, IDH2.s
6	fi	FISH (fluorescence in-situ hybridization)	6	cMET.fi, Her2.Neu.fi, del.1p.19q..fi, ALK_2p23.fi,
7	ci	chromogenic in situ hybridization	5	cMET.ci, EGFR.ci, Her2.ci, MDM2.ci, TOP2A.ci
8	r	restriction fragment length polymorphism	3	EGFR.r, EGFR_EX20ins.r, EGFR_T790M.r
9	fa	fragment analysis	2	EGFRvIII.fa, MSI.fa
10	fv	fusion variant	2	EGFR.fv, MET.fv
11	ffa	fusion or fragment analysis	1	EGFRvIII.ffa
12	hs	H score (immunohistochemistry score)	1	EGFR.hs
13	msi	microsatellite instability	1	
14	ps	pyrosequencing	1	MGMT.ps

Table 32: Method for molecular tests, sorted by number of tests

```
### number of NAs per test
na1 <- d1[, vapply(.SD, FUN=function(x) sum(is.na(x)), FUN.VALUE=1L),
          .SDcols=29:length(d1)]
xtable(t(as.matrix(summary(na1))),
       caption=paste0("All dichotomous molecular tests, n=",
                      length(mt1),
                      ", number of tests with result = not available"))
```

```
#### check for overlap between msi and msi via fa
t1 <- d1[, table(factor(MSI.fa, levels=0, labels="MSI.fa -ve"),
                 factor(msi, levels=c(0, 1), labels=c("MSI -ve", "MSI +ve")),
```

	Min.	1st Qu.	Median	Mean	3rd Qu.	Max.
1	134.00	573.00	602.00	626.47	602.00	1228.00

Table 33: All dichotomous molecular tests, n=1152, number of tests with result = not available

```
                  useNA="ifany")]
xtable(t1, caption="Comparison of methods used to assess MSI (microsatellite instabilit
```

	MSI -ve	MSI +ve	NA
MSI.fa..ve	13	0	5
NA.	631	5	575

Table 34: Comparison of methods used to assess MSI (microsatellite instability)

3.4 Get p values for contingency tables

```
### Fishers Exact Test - get p value
fetp <- function(x) tryCatch(
                        fisher.test(x)$p.value,
                        error=function(e) NaN)
####
#### this step can be slow, so we load the data below instead
#### uncomment below to perform the calculations
####
### pv1 = p values
### lt1 = list of contingency tables
## pv1 <- vector(mode="numeric", length=length(mt1))
## lt1 <- vector(mode="list", length=length(mt1))
## names(pv1) <- names(lt1) <- mt1
## for (i in seq_along(mt1)) {
##   if (interactive()) cat(paste0(i, " "))
##   lt1[[i]] <- d1[, table(gs, get(mt1[i]))]
##   pv1[i] <- fetp(d1[, table(gs, get(mt1[i]))])
## }
### order by p value
## pv1 <- pv1[order(pv1)]
## lt1 <- lt1[order(pv1)]
## save(pv1, file="pv1.dat")
## save(lt1, file="lt1.dat")
load("pv1.dat")
load("lt1.dat")
### all test results are -ve e.g.
## d1[, table(gs, get("Her2.ci"))]
stopifnot(length(pv1)==length(lt1))
length(pv1)
```

[1] 1156

```
### not done, or the results for the  MT were all +ve or -ve
length(which(is.nan(pv1)))
```

[1] 909

```
sum(pv1==1, na.rm=TRUE)
```

[1] 111

```
### or all inspect tables(in >= 1 case GB
if (interactive()) head(lt1[which(is.nan(pv1))])
###
```

3.5 MT not done

```
### lt1subset is used to drop elements from lt1
lt1subset <- lt1
length(drop1 <- which(sapply(lt1subset, function(x) length(x)==0)))

## [1] 4

cat("MT not done in either GS or GB: ",
    names(lt1subset)[drop1])

## MT not done in either GS or GB:  EGFR_T790M.c H3K36me3.i EGFR_T790M.n EGFR_Tertiary_

#### gene names
g1 <- unique(
     regmatches(names(lt1subset[drop1]),
                regexpr("[^_\\.]*", names(lt1subset[drop1]))))
u1 <- uid(esearch(term=g1, db="gene", sort="relevance", retmax=1))
#### print Hyperlink Reference to Gene database
hrg <- function(g, n)
    paste("\\href{http://www.ncbi.nlm.nih.gov/gene/", g, "}{", n, "}",
          sep="", collapse="")
## cat("\n", hrg(u1, g1), "\n")
### drop them
lt1subset <- lt1subset[-drop1]
```

3.6 MTs all either +ve or -ve

```
allPN1 <- which(sapply(lt1subset, function(x) length(x)==2))
### lt1APN = List of contingency Table which are
###  All Positive or Negative
###  this is a subset of lt1subset
length(lt1APN <- lt1subset[allPN1])
```

[1] 905

```
### MTs all +ve
allP1 <- which(sapply(lt1APN, function(x) colnames(x)=="1"))
length(allP1)
```

[1] 1

```
(ap1 <- names(lt1APN[allP1]))
```

[1] "EGFR.r"

```
g1 <- unique(
     regmatches(ap1,
                regexpr("[^_\\.]*", ap1)))
u1 <- uid(esearch(term=g1, db="gene", sort="relevance", retmax=1))
cat(hrg(u1, g1))
```

EGFR

```
### MTs all -ve
length(an1 <- lt1APN[-allP1])
```

[1] 904

```
if (interactive()){
    cat("\n All MTs -ve \n\n")
    cat(names(an1), fill=70, sep="\t")
}
c1 <- cbind2(
    unlist(lapply(an1, function(x) x["0", ])),
    unlist(lapply(an1, function(x) x["1", ])))
r1 <- rbindlist(list(
  c("GB", as.list(summary(c1[, 1]))),
  c("GS", as.list(summary(c1[, 2])))))
xtable(r1,
       caption="Pathology (GB vs. GS) X MTs all -ve; summary")
```

	V1	Min.	1st Qu.	Median	Mean	3rd Qu.	Max.
1	GB	0.00	603.00	603.00	573.69	629.00	1047.00
2	GS	0.00	24.00	26.00	23.95	27.00	47.00

Table 35: Pathology (GB vs. GS) X MTs all -ve; summary

```
### get protein names
an1 <-regmatches(names(an1),
                 regexpr(".+\\.", names(an1)))
an1 <- gsub("\\.$", "", an1)
length(pn2 <- unique(an1))
```

[1] 593

3.7 MTs all -ve

```
####
#### this step can be slow, so we load data below instead
####
### gan1 = genes (proteins) with all negative results
## invisible(
##     gan1 <- sapply(pn2, function(x)
##         esearch(term=x, db="gene", sort="relevance", retmax=1)))
## save(gan1, file="gan1.dat")
load("gan1.dat")
for (i in seq_along(gan1)) {
    if (i==1) cat("\\setstretch{1.5}")
    n1 <- gsub("_", "-", names(gan1)[i])
    u1 <- uid(gan1[[i]])
    if (!is.na(u1)) {
        cat(paste(hrg(u1, n1), " \\quad "))
    } else {
        cat(paste(n1, " \\quad "))
    }
    if (i==length(gan1)) cat("\\setstretch{1}")
}
```

Her2 MDM2 TOP2A X43352 ACSL3 ACSL6 ADGRA2 AFDN AFF1 AFF3 AFF4 ALDH2 ALK APC ARFRP1 ARHGAP26 ARID1A ARID2 ARNT ASPSCR1 ASXL1 ATF1 ATIC ATM ATR AURKA AURKB AXIN1 AXL BAP1 BARD1 BCL11A BCL2L11 BCL3 BCL7A BCL9 BCR BIRC3 BMPR1A BRCA1 BRCA2 BUB1B CACNA1D CAMTA1 CANT1 CARS CASP8 CBFB CBLB CCDC6 CCNB1IP1 CD74 CD79A CDC73 CDH11 CDK8 CDX2 CHEK1 CHEK2 CHN1 CIC CIITA CLP1 CLTCL1 CNBP CNTRL COPB1 CREB1 CREB3L1 CREB3L2 CREBBP CRTC1 CRTC3 CSF1R CSF3R CTCF CTLA4 CTNNA1 CTNNB1 CYLD CYP2D6 DAXX DDR2 DDX5 DICER1 EBF1 ECT2L ELL EML4 EMSY EP300 EPHA3 EPHB1 EPS15 ERBB2 ERBB4 ERCC2 ERCC3 ERCC4 ERG ESR1 ETV6 EWSR1 EXT1 EXT2 EZR FANCA FANCC FANCD2 FANCE FANCG FANCL FAS FBXO11 FBXW7 FCRL4 FGF19 FGFR1 FGFR1OP FGFR2 FGFR4 FHIT FLCN FLI1 FLT1 FLT4 FNBP1 FOXA1 FOXO1 FOXP1 FUBP1 FUS GATA3 GMPS GNA13 GNAQ GNAS GOLGA5 GOPC GPHN GRIN2A GSK3B H3F3B HERPUD1 HMGA1 HOOK3 HSP90AA1 IDH1 IDH2 IGF1R IL2 IL21R IL6ST IL7R IRF4 ITK JAK3 KEAP1 KIAA1549 KIF5B KMT2C KMT2D KNL1 KTN1 LCP1 LHFPL6 LIFR LRP1B MAF MAML2 MAP2K1 MAP2K2 MAP3K1 MDS2 MEF2B MEN1 MITF MLF1 MLH1 MLLT1 MLLT10 MLLT3 MLLT6 MRE11 MSH2 MSH6 MTOR MYB MYD88 MYH11 MYH9 NCKIPSD NCOA1 NCOA2 NCOA4 NF1 NF2 NFE2L2 NFKB2 NFKBIA NIN NPM1 NR4A3 NSD2 NSD3 NT5C2 NTRK1 NTRK2 NTRK3 NUP214 NUP93 NUP98 NUTM1 PALB2 PAX3 PAX5 PAX7 PBRM1 PBX1 PCM1 PCSK7 PDGFB PDGFRB PDK1 PER1 PICALM PIK3R1 PIK3R2 PML PMS2 POLE PPARG PRCC PRKAR1A PRRX1 PTCH1 PTEN PTPN11 PTPRC RABEP1 RAC1 RAD50 RAD51 RAD51B RAF1 RANBP17 RAP1GDS1 RARA RB1 REL RET RIC-TOR RMI2 ROS1 RPL22 RPL5 RPN1 RPTOR RUNX1 RUNx1T1 SDC4 SDHAF2 SDHC SET SETD2 SF3B1 SH2B3 SH3GL1 SMAD4 SMARCB1 SNX29 SOX10 SPECC1 SRGAP3 SRSF2 SS18 SS18L1 STAT3 STAT4 STAT5B STIL SUZ12 SYK TAF15 TCF12 TCF7L2 TET1 TET2 TFG TFRC TGFBR2

TLX1 TNFAIP3 TNFRSF14 TNFRSF17 TOP1 TP53 TPM3 TPM4 TRAF7 TRIM26 TRIM27 TRIP11 TSC1 TSC2 TSHR TTL U2AF1 VEGFA VEGFB VTI1A WDCP WISP3 WRN WWTR1 XPA XPC XPO1 YWHAE ZMYM2 ZNF217 ZNF384 ZNF521 ZNF703 MSI cMET Her2.Neu ALK-2p23 MET AKT3 BRD3 BRD4 ETV1 ETV4 ETV5 FGR INSR MAST1 MAST2 MSMB MUSK NOTCH1 NOTCH2 NRG1 NUMBL PDGFRA PIK3CA PKN1 PRKCA PRKCB RSPO2 RSPO3 TERT TFE3 TFEB THADA TMPRSS2 PD.L1-.22c3. X43348 X43349 ABI1 ABL1 ABL2 ACKR3 AKAP9 AKT1 AKT2 AMER1 Androgen-Receptor AR ARAF ARHGEF12 ATP1A1 ATP2B3 BCL10 BCL11B BCL2 BCL2L2 BCL6 BCOR BCORL1 BRIP1 BTG1 BTK C15orf65 CALR CARD11 CBFA2T3 CBL CBLC CCND1 CCND2 CCND3 CCNE1 CD274 CD79B CDK12 CDK4 CDK6 CDKN2B CDKN2C CEBPA CHCHD7 CHIC2 c.KIT CLTC CNOT3 COL1A1 COX6C CRKL CRLF2 DDB2 DDIT3 DDX10 DDX6 DEK DNM2 DNMT3A DOT1L EIF4A2 ELF4 ELK4 ELN EPHA5 ERBB3 ERC1 ERCC1 ERCC5 EZH2 FAM46C FANCF FEV FGF10 FGF14 FGF23 FGF3 FGF4 FGF6 FIP1L1 FLT3 FOXL2 FOXO4 FSTL3 GAS7 GATA1 GATA2 GID4 GNA11 GPC3 H3F3A HEY1 HGF HIP1 HIST1H3B HIST1H4I HLF HMGA2 HMGN2P46 HNRNPA2B1 HOXA11 HOXA13 HOXA9 HOXC11 HOXC13 HOXD11 HOXD13 HSP90AB1 IKBKE IKZF1 INHBA IRS2 JAK1 JAK2 JAZF1 JUN KAT6A KAT6B KCNJ5 KDM5A KDR KDSR KIT KLF4 KLHL6 KLK2 KMT2A LASP1 LCK LGR5 LMO1 LMO2 LPP LRIG3 LYL1 MAFB MALT1 MAP2K4 MAX MCL1 MDM4 MECOM MED12 MKL1 MLLT11 MN1 MNX1 MPL MSI2 MSN MTCP1 MUC1 MYC MYCL MYCN NACA NDRG1 NFIB NKX2.1 NONO NSD1 NUMA1 NUTM2B OLIG2 OMD P2RY8 PAFAH1B2 PAK3 PATZ1 PAX8 PDCD1 PDCD1LG2 PHF6 PHOX2B PIK3CG PIM1 PLAG1 PMS1 POU2AF1 POU5F1 PPP2R1A PRDM1 PRDM16 PRF1 PRKDC PSIP1 RAD21 RALGDS RBM15 RECQL4 RHOH RNF213 RNF43 RPL10 SBDS SDHD SETBP1 SFPQ SLC34A2 SLC45A3 SOCS1 SOX2 SPEN SPOP SRC SRSF3 SSX1 STAG2 STK11 TAL1 TAL2 TBL1XR1 TCEA1 TCF3 TCL1A TFPT THRAP3 TLX3 TPR TRIM33 TR-RAP UBR5 USP6 WAS WIF1 ZBTB16 ZNF331 ZRSR2 EGFR-EX20ins EGFR-T790M BRAF KRAS KRAS-G13D NRAS

3.8 MTs all +ve or -ve in either GB or GS but not both

```
#### back to lt1subset
### drop all -ve or +ve above
allPN1 <- which(sapply(lt1subset, function(x) length(x)==2))
length(lt1subset <- lt1subset[-allPN1])
```

[1] 247

```
### not done GS
length(ndGS1 <- which(sapply(lt1subset, function(x) x[2]==0 & x[4]==0)))
```

[1] 5

```
nd1 <- names(lt1subset[ndGS1])
cat("MT not available in any cases of GS: ", paste0(nd1, collapse=", "), "\n")
```

MT not available in any cases of GS: EGFR.ci, EGFR.hs, TrkA.B.C.i, BRCA1.s, BRCA2.s

```
### drop them
lt1subset <- lt1subset[-ndGS1]
### not done GB i.e. none
length(ndGB1 <- which(sapply(lt1subset, function(x) x[1]==0 & x[3]==0)))
```

[1] 0

```
### List of Tables with One Zero
ltoz1 <- vector(mode="list", length=4)
names(ltoz1) <- c("allGBpos", "allGSpos",
                  "allGBneg", "allGSneg")
for (i in seq_along(ltoz1)) {
    length(w1 <- which(sapply(lt1subset, function(x) x[i]==0)))
    if (interactive()) print(length(lt1subset[w1]))
    ltoz1[[i]] <- lt1subset[w1]
}
print(xtable(rbindlist(list(lapply(ltoz1, length))),
             caption="MTs where all results were +ve or -ve for either GB or GS"),
      include.rownames=FALSE)
```

allGBpos	allGSpos	allGBneg	allGSneg
0	0	1	192

Table 36: MTs where all results were +ve or -ve for either GB or GS

```
cat("MT negative in all cases of GB: ", names(ltoz1$allGBneg), "\n")
```

MT negative in all cases of GB: CALR.c

```
if (interactive()){
     cat("\n MT negative in all cases of GS: \n\n")
     cat(names(ltoz1$allGSneg), fill=70, sep="\t")
}
### get unique protein names
### angs1 = all negative in gliosarcoma
angs1 <- names(ltoz1$allGSneg)
angs1 <-regmatches(angs1,
                   regexpr(".+\\.", angs1))
angs1 <- gsub("\\.$", "", angs1)
### pn3 = protein names
length(pn3 <- unique(angs1))
```

[1] 172

```
####
#### this step can be slow, so we load data below instead
####
### gangs1 = genes (proteins) with all negative results in GS
## invisible(
##     gangs1 <- sapply(pn3, function(x)
##         esearch(term=x, db="gene", sort="relevance", retmax=1)))
```

```
## save(gangs1, file="gangs1.dat")
load("gangs1.dat")
stopifnot(!any(sapply(gangs1, function(x) is.na(uid(x)))))
cat("\n MT negative in all cases of GS: \n\n")
```

MT negative in all cases of GS:

```
for (i in seq_along(gangs1)) {
    if (i==1) cat("\\setstretch{1.5}")
    cat(paste(hrg(uid(gangs1[[i]]), names(gangs1[i])), "\\quad"))
    if (i==length(gangs1)) cat("\\setstretch{1}")
}
```

cMET ABL2 AKAP9 AKT2 AKT3 ARHGEF12 ATP1A1 BCL10 BCL6 BLM BRAF BRIP1 CARD11 CBFA2T3 CBL CCND1 CCND2 CCND3 CCNE1 CD274 CDK6 CDKN1B CDKN2A CHIC2 CLTC CRKL DDX10 DDX6 DEK DOT1L ELK4 EPHA5 ERBB3 ERC1 ERCC5 ETV1 ETV5 EZH2 FGF10 FGF14 FGF23 FGF3 FGF4 FGF6 FGFR3 FH FIP1L1 FLT3 GAS7 GID4 H3F3A Her2.Neu HGF HIP1 HMGA2 HNRNPA2B1 HSP90AB1 IKZF1 JAK1 JAK2 JAZF1 KDM5A KDR KIT KLHL6 KMT2A KRAS LCK LPP MALT1 MAP2K4 MCL1 MDM4 MNX1 MSI2 MYC MYCN NACA NFIB NOTCH2 NSD1 PDCD1 PDCD1LG2 PDGFRA PIK3CA PIM1 POT1 POU2AF1 PRDM1 PRDM16 PSIP1 RALGDS RBM15 RNF43 SBDS SDHB SDHD SETBP1 SLC34A2 SMAD2 SMARCE1 SMO SPEN SRSF3 STK11 SUFU TCF3 TFEB TPR TRIM33 TRRAP USP6 WIF1 WT1 ZNF331 EGFR MAML2 MET NTRK2 NTRK3 RELA ROS1 ER MLH1 MSH2 MSH6 PMS2 PR ARID1A ARID2 ATRX BAP1 BRCA1 CDC73 CDH1 CHEK1 CHEK2 CTNNB1 ERBB2 FANCC FBXW7 FGFR1 FLCN FOXO3 FUBP1 HNF1A HRAS IDH1 KDM5C KDM6A KMT2C MAP2K1 MITF MSI MTOR MUTYH NBN NOTCH1 NRAS PALB2 PBRM1 PDE4DIP PIK3R1 POLE RAD50 SMAD4 SMARCA4 SMARCB1 TSC1 TSC2 VHL IDH2

```
### we can confirm that all p values for these tests = 1
if (interactive()) lapply(ltoz1$allGSneg, fisher.test)
```

3.9 Summary of MTs

```
cat(paste("\n\\textbf{MT not performed in any cases of GS:} \n\n",
          paste(nd1, collapse=", "), "\n\n"))
```

MT not performed in any cases of GS:

EGFR.ci, EGFR.hs, TrkA.B.C.i, BRCA1.s, BRCA2.s

```
cat(paste("\n\\textbf{MTs all -ve in GB:}\n", names(ltoz1$allGBneg),
          "\n\n"))
```

MTs all -ve in GB: CALR.c

```
cat("\n\\textbf{MTs all -ve in GS:}\n\n")
```

MTs all -ve in GS:

```
if (interactive()) cat(names(ltoz1$allGSneg), fill=70, sep="\t")
```

3.10 p Values (FET)

```
load("pv1.dat")
sum(pv1 > (1 - 1e-3), na.rm=TRUE)

## [1] 194

sum(pv1 <= (1 - 1e-3), na.rm=TRUE)

## [1] 53

sum(pv1 < 0.1, na.rm=TRUE)

## [1] 14

sum(pv1 < 0.05, na.rm=TRUE)

## [1] 8
```

3.11 MTs with p value of 0.1 to 0.999

```
length(psv1 <- which(pv1 <= (1 - 1e-3) & pv1 >= 0.1))
```

[1] 39

```
psvn1 <- names(psv1)
if (interactive()) print(psvn1)
####
#### this step can be slow, so we load data below instead
####
### gpsv1 = genes (proteins) with significant values
## gpsv1 <-regmatches(psvn1,
##                     regexpr(".+\\.", psvn1))
## gpsv1 <- gsub("\\.$", "", gpsv1)
## invisible(
##     gpsv1 <- sapply(gpsv1, function(x)
##         esearch(term=x, db="gene", sort="relevance", retmax=1)))
## save(gpsv1, file="gpsv1.dat")
load("gpsv1.dat")
for (i in seq_along(gpsv1)) {
    if (i==1) cat("\\setstretch{1.5}")
    n1 <- gsub("_", "-", names(gpsv1)[i])
    u1 <- uid(gpsv1[[i]])
    if (!is.na(u1)) {
        cat(paste(hrg(u1, n1), " \\quad "))
    } else {
        cat(paste(n1, " \\quad "))
```

```
    }
    if (i==length(gpsv1)) cat("\\setstretch{1}")
}
```

LGR5 CIC PTEN BRCA2 ATRX MET PDGFRA MGMT TOP2A MSH6 KRAS RB1 MGMT EGFRvIII PIK3CA cMET T.M.L. LRIG3 MDM4 KIT TS APC TP53 SPARC-Polyclonal SETD2 PTEN TUBB3 TLE3 ATM BRAF CDK4 KDR CHIC2 FIP1L1 Androgen-Receptor EGFR MDM2 RRM1

3.12 MTs with p value of less than 0.1

```
### significant values
sv1 <- which(pv1 < 0.1)
### significant values - names
svn1 <- names(sv1)
####
#### this step can be slow, so we load data below instead
####
### gsv1 = genes (proteins) with significant values
## gsv1 <-regmatches(svn1,
##                   regexpr(".+\\.", svn1))
## gsv1 <- gsub("\\.$", "", gsv1)
## gsv1[c(2, 4)] <- c("PDL1", "PD1")
## invisible(
##     gsv1 <- sapply(gsv1, function(x)
##         esearch(term=x, db="gene", sort="relevance", retmax=1)))
## save(gsv1, file="gsv1.dat")
load("gsv1.dat")
paste0("Total number of tests with possibly significant values = ",
       sum(pv1 < 0.9999, na.rm=TRUE))
```

[1] "Total number of tests with possibly significant values = 53"

```
dt1 <- data.table(matrix(data="", nrow=length(svn1), ncol=11))
setnames(dt1, old=1:11, new=c("MT", "method", "link",
                              "p (FET)",
                              "nGS", "pGS",
                              "nGB", "pGB",
                              "OR", "95 CI", "ref"))
for (i in seq.int(gsv1)) {
    set(dt1, i=i, j="MT", value=hrg(uid(gsv1[[i]]), names(gsv1)[i]))
}
set(dt1, j="method", value=gsub(".+\\.+", "", svn1))
set(dt1, j="p (FET)", value=fc2(pv1[sv1]))
### ct1 = values from Contingency Tables for significant tests
ct1 <- matrix(unlist(lt1[svn1]), ncol=4, byrow=TRUE,
              dimnames=list(names(lt1[svn1]),
                            c("GB-", "GS-", "GB+", "GS+")))
set(dt1, j="nGS",
    value=paste(ct1[, "GS+"], "/", ct1[, "GS+"] + ct1[, "GS-"]))
set(dt1, j="nGB",
    value=paste(ct1[, "GB+"], "/", ct1[, "GB+"] + ct1[, "GB-"]))
set(dt1, j="pGS",
```

```
    value=fc2(100 * ct1[, "GS+"] / (ct1[, "GS+"] + ct1[, "GS-"])))
set(dt1, j="pGB",
    value=fc2(100 * ct1[, "GB+"] / (ct1[, "GB+"] + ct1[, "GB-"])))
set(dt1, j="OR", value=dt1[, fc2(as.numeric(pGS) / as.numeric(pGB))])
## 95% CI for OR
or1 <- sapply(lt1[svn1],
              FUN=function(x)
                  paste0(fc2(fisher.test(x)$conf.int), collapse=" - "))
set(dt1, j="95 CI", value=or1)
set(dt1, j="ref", value=rep("\\cite{}", nrow(dt1)))
dt1 <- dt1[order(as.numeric(dt1$OR), decreasing=TRUE), ]
options("xtable.include.rownames"=FALSE)
## write.csv(dt1, file="dt1.csv")
print(xtable(dt1,
             caption=
                 "Significant ($p < 0.1$) molecular tests; sorted by OR; 95\\% given fo
      sanitize.text.function = function(x){x})
```

MT	method	link	p (FET)	nGS	pGS	nGB	pGB	OR	95 CI	ref
CALR	c		0.038	1 / 24	4.2	0 / 603	0	Inf	0.64 - Inf	[]
NTRK1	f		0.074	1 / 22	4.5	1 / 563	0.18	25	0.33 - 2.1e+03	[]
LYL1	c		0.075	1 / 24	4.2	1 / 603	0.17	25	0.32 - 2e+03	[]
PTCH1	n		0.081	1 / 25	4	1 / 582	0.17	24	0.3 - 1.9e+03	[]
IDH2	n		0.08	1 / 27	3.7	1 / 631	0.16	23	0.3 - 1.9e+03	[]
PTPN11	n		0.08	3 / 47	6.4	21 / 1046	2	3.2	0.61 - 12	[]
NF1	n		0.019	8 / 25	32	81 / 587	14	2.3	1.1 - 7.5	[]
PDL1	i		0.0057	15 / 42	36	152 / 902	17	2.1	1.3 - 5.5	[]
PD1	i		0.014	20 / 26	77	211 / 408	52	1.5	1.2 - 9.6	[]
EGFRvIII	ffa		0.02	2 / 38	5.3	143 / 716	20	0.27	0.026 - 0.88	[]
EGFRvIII	fa		0.06	1 / 24	4.2	71 / 349	20	0.21	0.0041 - 1.1	[]
EGFR	c		0.00085	2 / 27	7.4	231 / 623	37	0.2	0.015 - 0.55	[]
EGFR	n		0.011	0 / 47	0	112 / 1044	11	0	0 - 0.69	[]
IDH1	n		0.046	0 / 47	0	87 / 1047	8.3	0	0 - 0.92	[]

Table 37: Significant ($p < 0.1$) molecular tests; sorted by OR; 95% given for OR.

3.13 Correlations

Here we check for significant correlations between some of the preceding tests.

```
d1[, ftable(gs, EGFR.c, EGFR.n, exclude=NaN)]

##            EGFR.n    0   1  NA
## gs EGFR.c
## 0  0               378  14   0
##    1               167  64   0
##    NA              387  34 137
## 1  0                25   0   0
##    1                 2   0   0
##    NA               20   0   1
```

```
d1[, ftable(gs, EGFRvIII.fa, EGFRvIII.ffa, exclude=NaN)]

##                 EGFRvIII.ffa   0   1  NA
## gs EGFRvIII.fa
## 0  0                          278   0   0
##    1                            0  63   8
##    NA                         295  80 457
## 1  0                           23   0   0
##    1                            0   1   0
##    NA                          13   1  10

d1[, table(EGFRvIII.fa, EGFRvIII.ffa, useNA="ifany")]

##             EGFRvIII.ffa
## EGFRvIII.fa   0   1 <NA>
##        0    301   0    0
##        1      0  64    8
##        <NA> 308  81  467

d1[, table(PTCH1.n, IDH1.n)]

##          IDH1.n
## PTCH1.n   0   1
##        0 551  53
##        1   2   0

d1[, table(PTCH1.n, IDH2.n)]

##          IDH2.n
## PTCH1.n   0   1
##        0 604   1
##        1   2   0
```

4 Additional modelling

4.1 Robustness

```
if (interactive()) print(lt1[svn1])
l1 <- vector(mode="list", length=length(lt1[svn1]))
names(l1) <- names((lt1[svn1]))
l2 <- vector(mode="list", length=8L)
for (i in seq_along(l1)) {
    ## contingency table
    ct2 <- lt1[svn1][[i]]
    for (j in 1:4) {
        ct3 <- ct2
        ct3[j] <- ct3[j] + 1
        l2[[j]]$ct <- ct3
        l2[[j]]$p <- fetp(ct3)
    }
```

```
    for (j in 1:4) {
        ct3 <- ct2
        ct3[j] <- ct3[j] - 1
        l2[[j+4]]$ct <- ct3
        l2[[j+4]]$p <- fetp(ct3)
    }
    l1[[i]] <- l2
}
if (interactive()) print(l1)
pv2 <- lapply(l1,
              function(x) unname(unlist(x)[names(unlist(x))=="p"]))
dt2 <- data.table("test"=names(pv1[svn1]),
                  "p"=pv1[sv1],
                  "p05"=ifelse(pv1[svn1] < 0.05, "*", ""),
                  "r1"=vapply(pv2,
                              FUN=function(x)
                                  ifelse(all(x < 0.1, na.rm=TRUE),
                                         "*", ""),
                              FUN.VALUE=""),
                  "r05"=vapply(pv2,
                              FUN=function(x)
                                  ifelse(all(x < 0.05, na.rm=TRUE),
                                         "*", ""),
                              FUN.VALUE=""))
dt2 <- dt2[order(dt2[, "p"]), ]
setnames(dt2, c("Test", "p (FET)", "p < 0.05",
                    "robust p < 0.1", "robust p < 0.05"))
print(xtable(dt2,
             digits=4,
             caption="Robustness of p values"))
```

Test	p (FET)	$p < 0.05$	robust $p < 0.1$	robust $p < 0.05$
EGFR.c	0.0009	*	*	*
PD.L1_.SP142..i	0.0057	*	*	*
EGFR.n	0.0113	*	*	
PD.1.i	0.0144	*	*	*
NF1.n	0.0191	*	*	
EGFRvIII.ffa	0.0202	*	*	
CALR.c	0.0383	*		
IDH1.n	0.0463	*		
EGFRvIII.fa	0.0598			
NTRK1.f	0.0739			
LYL1.c	0.0751			
PTPN11.n	0.0801			
IDH2.n	0.0804			
PTCH1.n	0.0807			

Table 38: Robustness of p values

```
## write.csv(dt2, file="dt2.csv")
```

4.2 Recursive partitioning

```
### reload data
stopifnot(dim(d1 <- data.table(
                  read.gnumeric.sheet("./gb-gs.gnumeric",
                                      head=TRUE,
                                      sheet.name="data1"))) == c(1493, 1184))
### drop cases of GB which were biopsy-only
d1 <- d1[!(d1$gs==0 & d1$bx==1), ]
### add back optimal cutpoint for tmb
set(d1, j="tmbGEq35",
    value=as.numeric(d1$tmb) >= cut1)
### drop the following variables
suppressWarnings(
    for (i in c("id", "deId", "year", "inst", "state", "ageR",
                "lat", "s2", "s3", "tmb",
                "pOF", "lin", "gsf")){
        set(d1, j=i, value=NULL)
    }
)
r1 <- rpart(gs ~ ., data=d1,
            model=TRUE, method="class", control=rpart.control(minsplit=1))
### this has too many splits, as we can see:
rpart.plot(r1, snip=interactive(), cex=0.75)
### drop age and tumor site from the model
r2 <- rpart(gs ~ ., data=d1[, !c("age", "s1")],
            model=TRUE, method="class", control=rpart.control(minsplit=2))
### very little improvement
rpart.plot(r2, snip=interactive(), cex=0.75)
r3 <- rpart(gs ~ ., data=d1,
            model=TRUE, method="class", control=rpart.control(minsplit=3))
rpart.plot(r3,
           varlen=10, faclen=10, type=1, extra=106, under=TRUE, yesno=TRUE)
### take the best predictors seen in model r2 above
r4 <- rpart(gs ~ CALR.c + NTRK1.f + PD.L1_.SP142..i + bx + gen
          + IDH2.n + BRCA2.n, data=d1,
            model=TRUE, method="class", control=rpart.control(minsplit=1))
rpart.plot(r4, snip=interactive(), cex=0.75)
```

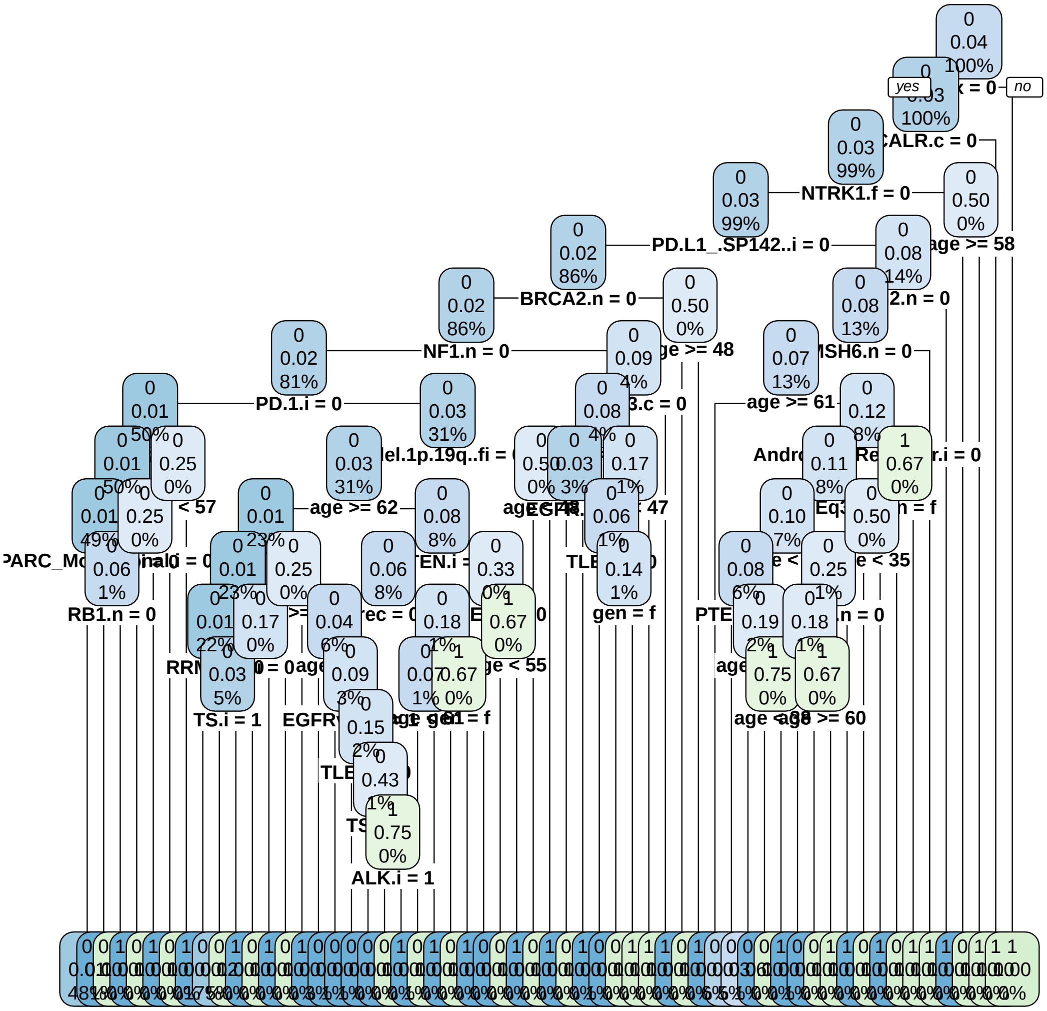
yes
no
CALR.c = 0
NTRK1.f = 0
PD.L1_.SP142..i = 0
BRCA2.n = 0
NF1.n = 0
PD.1.i = 0
age >= 58
age >= 48
MSH6.n = 0
age >= 61
age >= 62
RB1.n = 0
TS.i = 1
ALK.i = 1
gen = f
age < 55
age < 35
age >= 60

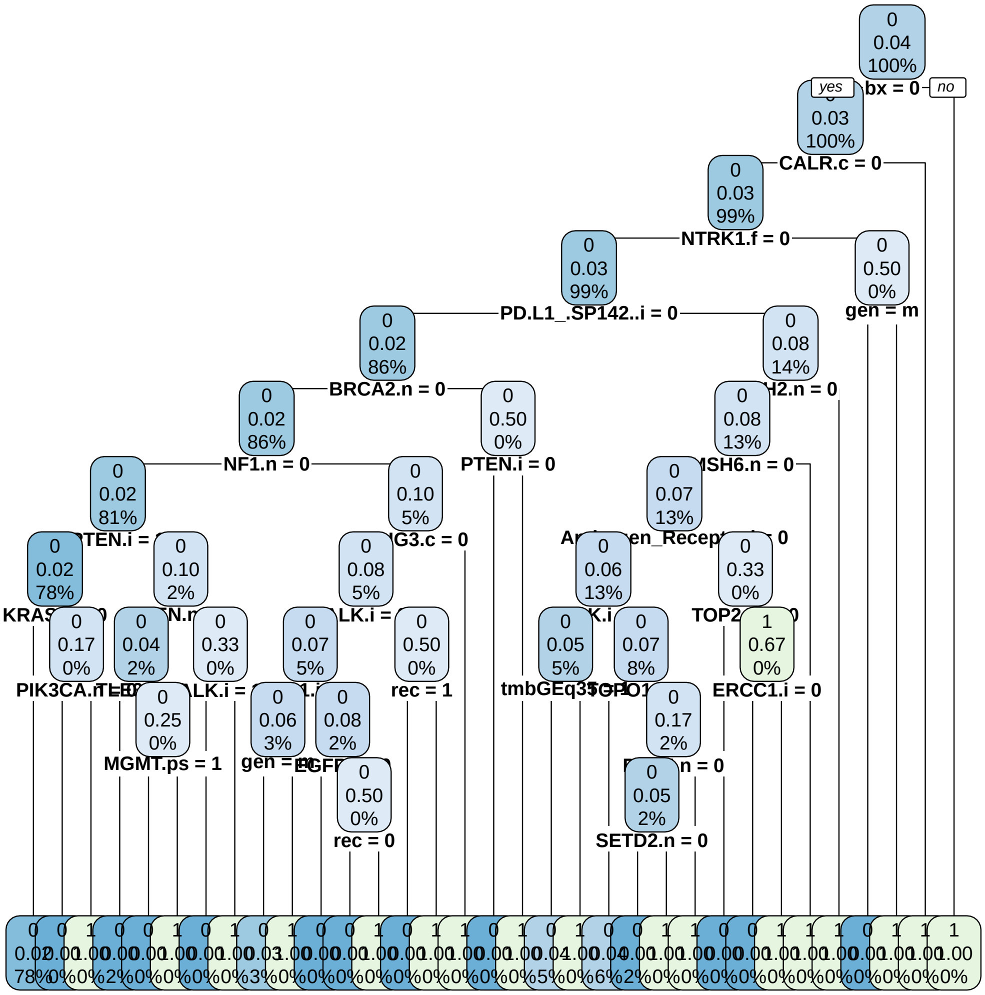
yes
no
CALR.c = 0
NTRK1.f = 0
PD.L1_.SP142..i = 0
BRCA2.n = 0
NF1.n = 0
PTEN.i = 0
gen = m
MGMT.ps = 1
rec = 1
rec = 0
SETD2.n = 0
ERCC1.i = 0

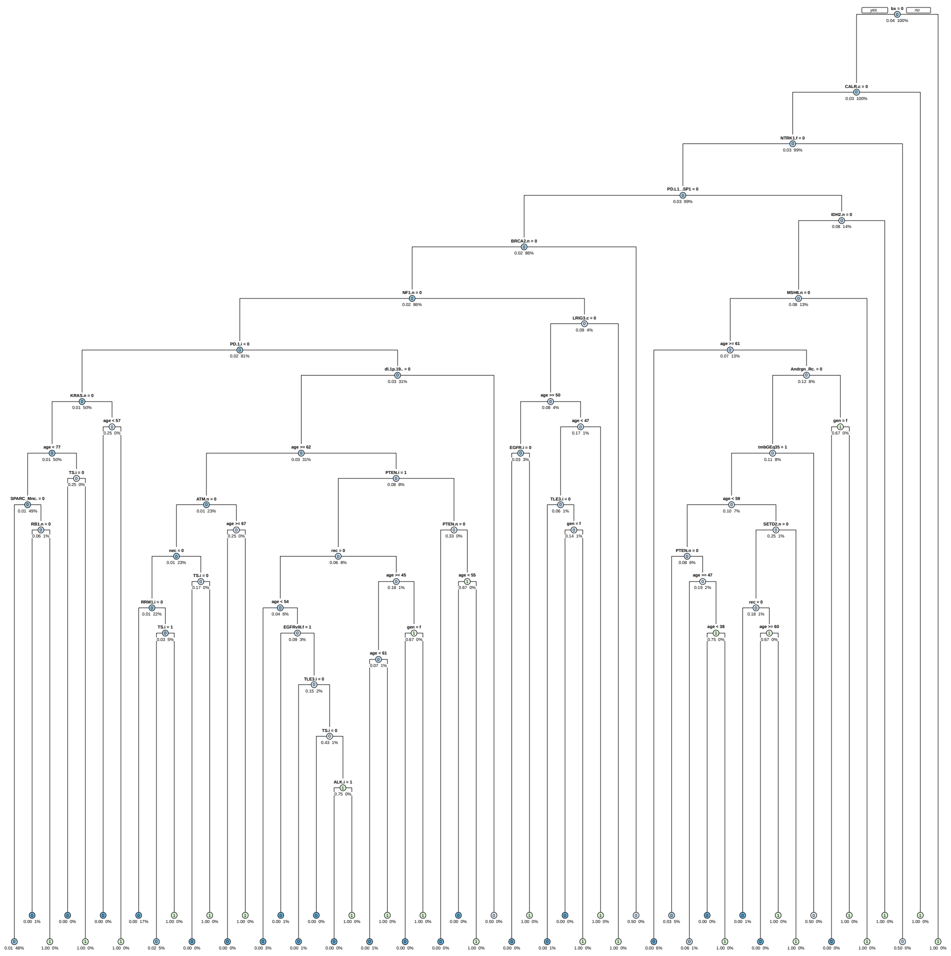

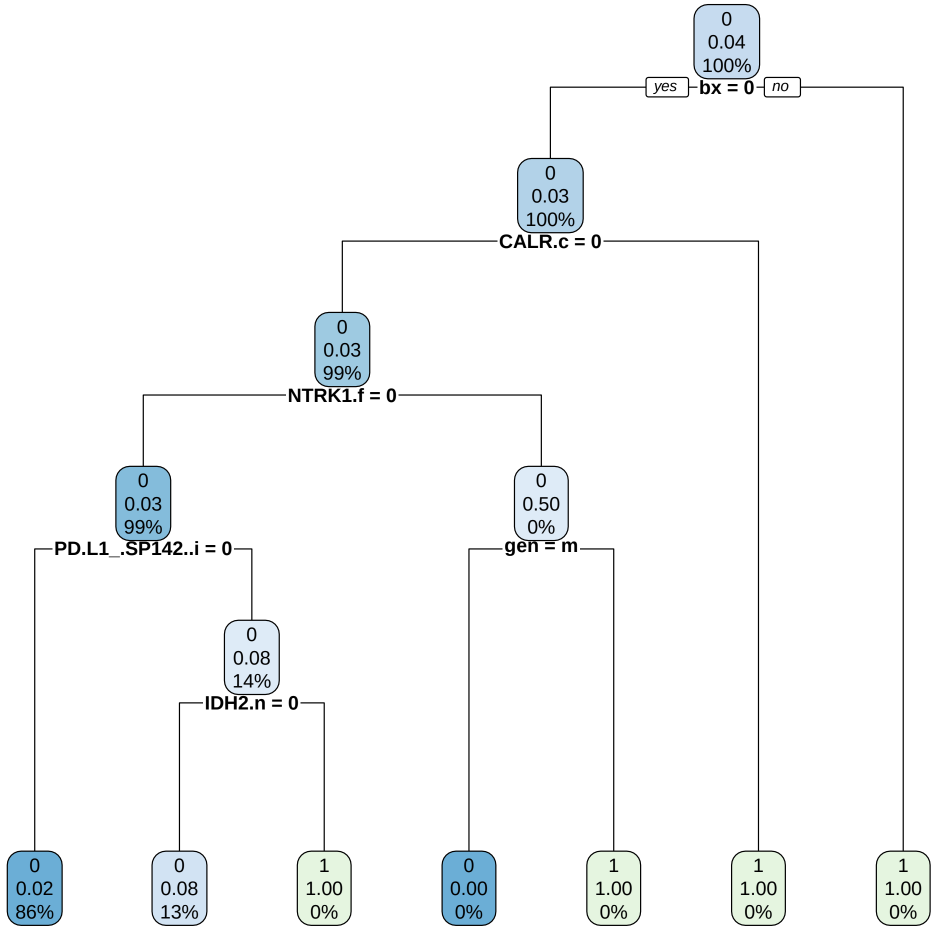

4.3 Logistic regression

```
options("xtable.include.rownames"=TRUE)
cap <- function(x){
    r1 <- regmatches(deparse(x$call),
                     regexpr("formula.+?,.", deparse(x$call)))
    sub("~", "by", x=r1)}
glm1 <- glm(gs ~ age, data=d1,
            family=binomial(link="logit"))
xtable(s1 <- summary(glm1),
       caption=cap(s1))
```

```
### significant values - names
xtable(s1 <- summary(glm(gs ~ 1, data=d1[, .SD, .SDcols=c("gs", svn1)])),
       caption="formula = gs by intercept-only")
```

	Estimate	Std. Error	z value	Pr(>|z|)
(Intercept)	-2.3720	0.5734	-4.14	0.0000
age	-0.0150	0.0103	-1.46	0.1436

Table 39: formula = gs by age,

	Estimate	Std. Error	t value	Pr(>|t|)
(Intercept)	0.0391	0.0055	7.06	0.0000

Table 40: formula = gs by intercept-only

```
xtable(s1 <- summary(glm(gs ~ .,
                         data=d1[, .SD, .SDcols=c("gs", svn1)],
                         family=binomial(link="logit"))),
       caption="Multi-variable model")
```

	Estimate	Std. Error	z value	Pr(>|z|)
(Intercept)	-24.5661	226917.1850	-0.00	0.9999
EGFR.c	-0.0000	173310.8628	-0.00	1.0000
PD.L1_.SP142..i	0.0000	253701.1255	0.00	1.0000
PD.1.i	-0.0000	185277.1057	-0.00	1.0000
NF1.n	0.0000	131010.6978	0.00	1.0000
EGFRvIII.ffa	0.0000	173310.8628	0.00	1.0000
IDH1.n	-0.0000	262021.3957	-0.00	1.0000

Table 41: Multi-variable model

```
### in case we want to store results of xtables below
l1 <- vector(mode="list", length=length(svn1))
### uni-variable models
suppressWarnings(
    for (i in seq_along(svn1)){
        l1[[i]] <-  xtable(summary(glm(gs ~ get(svn1[i]),
                                       data=d1[, .SD, .SDcols=c("gs", svn1)],
                                       family=binomial(link="logit"))))
    }
)
r1 <-rbindlist(l1)
### inverse of logit is probability
set(r1, j="OR", value=glm1$family$linkinv(r1$Estimate))
### convert this to Odds Ratio
set(r1, j="OR", value=OR(r1$OR))
r1 <- rbindlist(list(lapply(r1, fc2)))
set(r1, j="model", value="Intercept")
set(r1, i=seq.int(from=2L, to=28L, by=2L), j="model", value=svn1)
setcolorder(r1, neworder=6L)
colnames(r1)[5] <- "p"
sort1 <- r1[seq.int(from=2L, to=28L, by=2L), 2*order(p)]
sort2 <- sort1 - 1
r1 <- r1[c(rbind(sort2, sort1)), ]
```

```
xtable(r1,
       caption="Uni-variable models; sorted by p value for variable; intercept term app
```

	model	Estimate	Std. Error	z value	p	OR
1	Intercept	-3.3	0.2	-17	1.4e-64	0.036
2	PD.L1_.SP142..i	1	0.33	3	0.0025	2.7
3	Intercept	-2.8	0.21	-13	1.3e-40	0.064
4	EGFR.c	-2	0.74	-2.7	0.0069	0.14
5	Intercept	-3.4	0.25	-14	4.3e-43	0.034
6	NF1.n	1.1	0.45	2.4	0.015	2.9
7	Intercept	-3.5	0.41	-8.4	3.6e-17	0.03
8	PD.1.i	1.1	0.48	2.4	0.017	3.1
9	Intercept	-3.3	0.22	-15	1.7e-49	0.037
10	NTRK1.f	3.3	1.4	2.3	0.022	27
11	Intercept	-3.3	0.21	-15	2.7e-53	0.038
12	LYL1.c	3.3	1.4	2.3	0.022	26
13	Intercept	-3.2	0.2	-16	4e-57	0.041
14	IDH2.n	3.2	1.4	2.2	0.026	24
15	Intercept	-3.2	0.21	-15	7.8e-53	0.041
16	PTCH1.n	3.2	1.4	2.2	0.026	24
17	Intercept	-2.8	0.17	-16	2.3e-58	0.063
18	EGFRvIII.ffa	-1.5	0.73	-2.1	0.04	0.22
19	Intercept	-3.1	0.15	-20	6.1e-93	0.043
20	PTPN11.n	1.2	0.64	1.9	0.059	3.3
21	Intercept	-2.5	0.22	-11	1.5e-30	0.083
22	EGFRvIII.fa	-1.8	1	-1.7	0.085	0.17
23	Intercept	-3	0.15	-20	8e-89	0.05
24	EGFR.n	-16	6.2e+02	-0.025	0.98	1.7e-07
25	Intercept	-3.3	0.21	-15	2.4e-53	0.038
26	CALR.c	18	8.8e+02	0.02	0.98	5.6e+07
27	Intercept	-3	0.15	-20	1.1e-90	0.049
28	IDH1.n	-16	7e+02	-0.022	0.98	1.8e-07

Table 42: Uni-variable models; sorted by p value for variable; intercept term appears first followed by the variable

```
### CIs for OR
for (i in seq_along(svn1)){
    l1[[i]] <-  OR(glm(gs ~ get(svn1[i]),
                       data=d1[, .SD, .SDcols=c("gs", svn1)],
                       family=binomial(link="logit")))
}
names(l1) <- svn1
## l1
### 3x most significant, all p < 0.01
xtable(r1[seq.int(2, 6, by=2), c("model", "p", "OR")],
       caption="Uni-variable models; 3 most significant variables")
```

```
### 3 most significant variables from the above
xtable(s1 <- summary(glm(gs ~ PD.L1_.SP142..i + EGFR.c + PD.1.i,
                         data=d1[, .SD, .SDcols=c("gs", svn1)],
```

	model	p	OR
1	PD.L1_.SP142..i	0.0025	2.7
2	EGFR.c	0.0069	0.14
3	NF1.n	0.015	2.9

Table 43: Uni-variable models; 3 most significant variables

```
                        family=binomial(link="logit"))),
       caption="GS by PD.L1.i + EGFR.c + PD.1.i")
```

	Estimate	Std. Error	z value	Pr(>|z|)
(Intercept)	-3.0529	0.7345	-4.16	0.0000
PD.L1_.SP142..i	-0.6147	0.8084	-0.76	0.4470
EGFR.c	-1.3671	0.7871	-1.74	0.0824
PD.1.i	1.4937	0.7910	1.89	0.0590

Table 44: GS by PD.L1.i + EGFR.c + PD.1.i

```
### 2 most significant variables from the above
### PD.1.i remains significant
(x1 <- xtable(s1 <- summary(glm(gs ~ EGFR.c + PD.1.i,
                              data=d1[, .SD, .SDcols=c("gs", svn1)],
                              family=binomial(link="logit"))),
             caption="GS by EGFR.c + PD.1.i"))
```

	Estimate	Std. Error	z value	Pr(>|z|)
(Intercept)	-3.1006	0.7324	-4.23	0.0000
EGFR.c	-1.3600	0.7860	-1.73	0.0836
PD.1.i	1.4151	0.7854	1.80	0.0716

Table 45: GS by EGFR.c + PD.1.i

```
r1 <- rbindlist(list(x1))
set(r1, j="OR", value=glm1$family$linkinv(r1$Estimate))
### convert this to Odds Ratio
set(r1, j="OR", value=OR(r1$OR))
r1 <- rbindlist(list(lapply(r1, fc2)))
set(r1, j="variable", value=rownames(x1))
setcolorder(r1, neworder=6L)
colnames(r1)[5] <- "p"
xtable(r1,
       caption="Multi-variable models; 2 most significant variables")
```

```
### in more detail
g1 <- glm(gs ~ EGFR.c + PD.1.i,
          data=d1[, .SD, .SDcols=c("gs", svn1)],
          family=binomial(link="logit"))
```

	variable	Estimate	Std. Error	z value	p	OR
1	(Intercept)	-3.1	0.73	-4.2	2.3e-05	0.045
2	EGFR.c	-1.4	0.79	-1.7	0.084	0.26
3	PD.1.i	1.4	0.79	1.8	0.072	4.1

Table 46: Multi-variable models; 2 most significant variables

```
xtable(OR(g1, what="all")[, -c("logit")],
       caption="Best two-variable model; Probabilities and ORs for all covariate groups
```

	model	P	lowP	upP	OR	lowOR	upOR
1	(Intercept)	0.04	0.01	0.16	0.05	0.01	0.19
2	EGFR.c	0.20	0.06	0.52	0.26	0.06	1.08
3	PD.1.i	0.80	0.49	0.95	4.12	0.98	17.32
4	(Intercept)+EGFR.c	0.01	0.00	0.05	0.01	0.00	0.05
5	(Intercept)+PD.1.i	0.16	0.04	0.44	0.19	0.04	0.78
6	EGFR.c+PD.1.i	0.51	0.20	0.82	1.06	0.25	4.45
7	(Intercept)+EGFR.c+PD.1.i	0.05	0.01	0.17	0.05	0.01	0.20

Table 47: Best two-variable model; Probabilities and ORs for all covariate groups, with 95% CIs (lower, upper)

5 Comparison to existing literature

This supports the 'Discussion' section in the main article.

```
set(d1, j="gsf", value=factor(d1$gs, levels=c(0, 1),
                              labels=c("GB", "GS")))
### ptv = print tables for a vector
ptv <- function(x){
    for (i in seq.int(x)){
    t1 <- d1[, table(gsf, factor(get(x[i])))]
    print(xtable(t1,
                 caption=(paste0(x[i], ", p (FET) = ", fc2(fetp(t1))))),
          table.placement="H")
    }}
### Wojtas et al.
### PTEN
v1 <- grep("PTEN", names(d1), value=TRUE)
ptv(v1)
```

	0
GB	603
GS	24

Table 48: PTEN.c, p (FET) = NaN

	0	1
GB	31	590
GS	4	33

Table 49: PTEN.i, p (FET) = 0.13

	0	1
GB	762	210
GS	33	12

Table 50: PTEN.n, p (FET) = 0.46

```
### PI3K
v1 <- grep("PIK3", names(d1), value=TRUE)
ptv(v1)
```

	0	1
GB	600	3
GS	24	0

Table 51: PIK3CA.c, p (FET) = 1

	0
GB	603
GS	24

Table 52: PIK3R1.c, p (FET) = NaN

	0
GB	603
GS	24

Table 53: PIK3R2.c, p (FET) = NaN

	0
GB	180
GS	2

Table 54: PIK3CA.f, p (FET) = NaN

	0	1
GB	943	98
GS	45	2

Table 55: PIK3CA.n, p (FET) = 0.31

	0
GB	629
GS	27

Table 56: PIK3CG.n, p (FET) = NaN

	0	1
GB	602	14
GS	27	0

Table 57: PIK3R1.n, p (FET) = 1

	0
GB	530
GS	22

Table 58: PIK3R2.n, p (FET) = NaN

	0
GB	5
GS	0

Table 59: PIK3CA.s, p (FET) = NaN

```
### BRAF
v1 <- grep("BRAF", names(d1), value=TRUE)
ptv(v1)
```

	0	1
GB	602	1
GS	24	0

Table 60: BRAF.c, p (FET) = 1

	0	1
GB	501	2
GS	16	0

Table 61: BRAF.f, p (FET) = 1

	0	1
GB	1031	16
GS	46	1

Table 62: BRAF.n, p (FET) = 0.53

	0
GB	6
GS	0

Table 63: BRAF.s, p (FET) = NaN

```
### Cho et al.
### TP53
v1 <- grep("TP53", names(d1), value=TRUE)
ptv(v1)
```

	0
GB	603
GS	24

Table 64: TP53.c, p (FET) = NaN

	0	1
GB	699	342
GS	29	18

Table 65: TP53.n, p (FET) = 0.43

```
### FGFR
v1 <- grep("FGFR", names(d1), value=TRUE)
ptv(v1)
```

	0
GB	623
GS	27

Table 66: FGFR1.c, p (FET) = NaN

	0
GB	603
GS	24

Table 67: FGFR1OP.c, p (FET) = NaN

	0
GB	623
GS	27

Table 68: FGFR2.c, p (FET) = NaN

	0	1
GB	620	3
GS	27	0

Table 69: FGFR3.c, p (FET) = 1

	0
GB	603
GS	24

Table 70: FGFR4.c, p (FET) = NaN

	0
GB	180
GS	2

Table 71: FGFR1.f, p (FET) = NaN

	0
GB	180
GS	2

Table 72: FGFR2.f, p (FET) = NaN

	0	1
GB	174	6
GS	2	0

Table 73: FGFR3.f, p (FET) = 1

	0	1
GB	1043	2
GS	47	0

Table 74: FGFR1.n, p (FET) = 1

	0
GB	599
GS	27

Table 75: FGFR1OP.n, p (FET) = NaN

	0
GB	1035
GS	47

Table 76: FGFR2.n, p (FET) = NaN

	0	1
GB	630	1
GS	27	0

Table 77: FGFR3.n, p (FET) = 1

	0
GB	631
GS	27

Table 78: FGFR4.n, p (FET) = NaN

```
### others from Cho et al.
v0 <- c("RASGRF", "COL5", "ITGB", "PAK3", "CACNA", "PLCB", "ITPR")
v1 <- unlist(sapply(v0, FUN=function(x) grep(x, names(d1), value=TRUE)))
ptv(v1)
```

	0
GB	481
GS	25

Table 79: PAK3.n, p (FET) = NaN

	0
GB	603
GS	24

Table 80: CACNA1D.c, p (FET) = NaN

	0
GB	628
GS	27

Table 81: CACNA1D.n, p (FET) = NaN

```
### Lowder et al.
(v1 <- grep("WNT", names(d1), value=TRUE))
```

character(0)

```
v1 <- grep("NFKB", names(d1), value=TRUE)
ptv(v1)
```

	0
GB	603
GS	24

Table 82: NFKB2.c, p (FET) = NaN

	0
GB	623
GS	27

Table 83: NFKBIA.c, p (FET) = NaN

	0
GB	523
GS	25

Table 84: NFKB2.n, p (FET) = NaN

	0
GB	623
GS	27

Table 85: NFKBIA.n, p (FET) = NaN

```
v1 <- grep("CDKN2A", names(d1), value=TRUE)
ptv(v1)
```

	0	1
GB	620	3
GS	27	0

Table 86: CDKN2A.c, p (FET) = 1

	0	1
GB	405	16
GS	23	0

Table 87: CDKN2A.n, p (FET) = 1

```
v1 <- grep("HOX", names(d1), value=TRUE)
ptv(v1)
```

	0
GB	608
GS	27

Table 88: HOXA11.n, p (FET) = NaN

	0
GB	630
GS	27

Table 89: HOXA13.n, p (FET) = NaN

	0
GB	631
GS	27

Table 90: HOXA9.n, p (FET) = NaN

	0
GB	625
GS	27

Table 91: HOXC11.n, p (FET) = NaN

	0
GB	625
GS	27

Table 92: HOXC13.n, p (FET) = NaN

	0
GB	614
GS	26

Table 93: HOXD11.n, p (FET) = NaN

	0
GB	631
GS	27

Table 94: HOXD13.n, p (FET) = NaN

	0
GB	631
GS	27

Table 95: PHOX2B.n, p (FET) = NaN

6 R citations

This is used to generate the citations to all of the R packages used in generating this file.

```
if (interactive()) print(.packages())
### vector for citations
vc1 <- unlist(sapply(X=rev(.packages()),
                     FUN=function(x) utils::toBibtex(utils::citation(x))))
vc1 <- vc1[!vc1=='']
### names for citations
n1 <- vc1[which(grepl(pattern='.year', x=vc1))]
n1  <- paste0(
    sub(pattern="\\.year$", replacement="", x=names(n1)),
    regmatches(x=n1, m=regexpr(pattern='[0123456789]+', text=n1)))
### group as list
l1 <- vector(mode='list', length=length(n1))
names(l1) <- n1
end1 <- which(grepl(pattern='\\}$', x=vc1))
### remove trailing comma from last item in .bib entry, if necessary
vc1[end1-1] <- sub(pattern=",$", replacement="", x=vc1[end1-1])
end1 <- c(0, end1)
for (i in seq.int(length.out=length(l1)))
    l1[[i]] <- unname(vc1[seq.int(from=(end1[i] + 1L), to=end1[i+1])])
l1 <- l1[!duplicated(l1)]
### add a handle, if necessary
for (i in seq.int(length.out=length(l1)))
    if (grepl(pattern='\\{,$', x=l1[[i]][1]))
         l1[[i]][1] <- sub(pattern=',$',
                           replacement=paste0(names(l1[i]), ','),
                           x=l1[[i]][1])
bib1 <- readLines('gs-gb.bib')
for (i in seq.int(length.out=length(l1)))
    if (!(l1[[i]][1] %in% bib1))
        write(x=l1[[i]], file='gs-gb.bib', append=TRUE)
```

7 Data

```
if (interactive()) dput(d1)
```

References

[1] Karoly Antal. *gnumeric: Read Data from Files Readable by 'gnumeric'*, 2017. R package version 0.7-8.

[2] David B. Dahl, David Scott, Charles Roosen, Arni Magnusson, and Jonathan Swinton. *xtable: Export Tables to LaTeX or HTML*, 2019. R package version 1.8-4.

[3] Chris Dardis. *LogisticDx: Diagnostic Tests for Models with a Binomial Response*, 2015. R package version 0.2.

[4] Paolo Di Lorenzo. *usmap: US Maps Including Alaska and Hawaii*, 2020. R package version 0.5.1.

[5] Matt Dowle and Arun Srinivasan. *data.table: Extension of 'data.frame'*, 2020. R package version 1.13.2.

[6] Duncan Temple Lang and the CRAN Team. *XML: Tools for Parsing and Generating XML Within R and S-Plus*, 2019. R package version 3.98-1.20.

[7] Stephen Milborrow. *rpart.plot: Plot 'rpart' Models: An Enhanced Version of 'plot.rpart'*, 2020. R package version 3.0.9.

[8] R Core Team. *R: A Language and Environment for Statistical Computing*. R Foundation for Statistical Computing, Vienna, Austria, 2020.

[9] Gerhard Schöfl. *reutils: Talk to the NCBI EUtils*, 2016. R package version 0.2.3.

[10] Terry Therneau and Beth Atkinson. *rpart: Recursive Partitioning and Regression Trees*, 2019. R package version 4.1-15.

[11] Christian Thiele. *cutpointr: Determine and Evaluate Optimal Cutpoints in Binary Classification Tasks*, 2020. R package version 1.0.32.

[12] Hadley Wickham. *ggplot2: Elegant Graphics for Data Analysis*. Springer-Verlag New York, 2016.

[13] Yihui Xie. knitr: A comprehensive tool for reproducible research in R. In Victoria Stodden, Friedrich Leisch, and Roger D. Peng, editors, *Implementing Reproducible Computational Research*. Chapman and Hall/CRC, 2014. ISBN 978-1466561595.

[14] Yihui Xie. *Dynamic Documents with R and knitr*. Chapman and Hall/CRC, Boca Raton, Florida, 2nd edition, 2015. ISBN 978-1498716963.

[15] Yihui Xie. *knitr: A General-Purpose Package for Dynamic Report Generation in R*, 2020. R package version 1.30.
